# Novel Urinary Biomarkers For Improved Prediction Of Progressive eGFR Loss In Early Chronic Kidney Disease Stages And In High Risk Individuals Without Chronic Kidney Disease

**DOI:** 10.1038/s41598-018-34386-8

**Published:** 2018-10-29

**Authors:** María E. Rodríguez-Ortiz, Claudia Pontillo, Mariano Rodríguez, Petra Zürbig, Harald Mischak, Alberto Ortiz

**Affiliations:** 1 Instituto de Investigación Sanitaria Fundación Jiménez Díaz, Fundación Renal Iñigo Álvarez de Toledo, Universidad Autónoma de Madrid. REDinREN, Avda. Reyes Católicos, 2, 28040 Madrid, Spain; 2 Mosaiques Diagnostics GmbH, Rotenburger Str. 20, 30659 Hannover, Germany; 3 Nephrology Service, Reina Sofía University Hospital/University of Córdoba. Instituto Maimónides de Investigación Biomédica de Córdoba (IMIBIC). Avda. Menéndez Pidal, S/N. 14004, Córdoba(Spain). REDinREN, Madrid, Spain; 4 Institute of Cardiovascular and Medical Sciences, 126 University Pl, G12 8TA Glasgow, United Kingdom

## Abstract

Chronic kidney disease is associated with increased risk of CKD progression and death. Therapeutic approaches to limit progression are limited. Developing tools for the early identification of those individuals most likely to progress will allow enriching clinical trials in high risk early CKD patients. The CKD273 classifier is a panel of 273 urinary peptides that enables early detection of CKD and prognosis of progression. We have generated urine capillary electrophoresis-mass spectrometry-based peptidomics CKD273 subclassifiers specific for CKD *s*tages to allow the early identification of patients at high risk of CKD progression. In the validation cohort, the CKD273 subclassifiers outperformed albuminuria and CKD273 classifier for predicting rapid loss of eGFR in individuals with baseline eGFR > 60 ml/min/1.73 m^2^. In individuals with eGFR > 60 ml/min/1.73 m^2^ and albuminuria <30 mg/day, the CKD273 subclassifiers predicted rapid eGFR loss with AUC ranging from 0.797 (0.743–0.844) to 0.736 (0.689–0.780). The association between CKD273 subclassifiers and rapid progression remained significant after adjustment for age, sex, albuminuria, DM, baseline eGFR, and systolic blood pressure. Urinary peptidomics CKD273 subclassifiers outperformed albuminuria and CKD273 classifier for predicting the risk of rapid CKD progression in individuals with eGFR > 60 ml/min/1.73 m^2^. These CKD273 subclassifiers represented the earliest evidence of rapidly progressive CKD in non-albuminuric individuals with preserved renal function.

## Introduction

Chronic kidney disease (CKD) is defined as the presence of either evidence of kidney damage (pathological albuminuria, abnormal urinary sediment of kidney origin, abnormal imaging studies or other criteria) or a glomerular filtration rate (GFR) below 60 ml/min/1.73 m^2^ persisting for 3 months or longer^1^. CKD is stratified in categories G1 to G5 according to GFR, and A1 to A3 according to albuminuria^1^. The prevalence of CKD is estimated at 10% of the global adult population^2^. The relevance of CKD is based on the higher risk of progression to end-stage renal disease requiring dialysis, as well as on the higher risk of all-cause and cardiovascular death^1,3–5^. Indeed, CKD was among the fastest growing causes of death worldwide^6^. This is in part due to the paucity of tools for early identification of patients at high risk for CKD progression and to the poor therapeutic armamentarium that is not optimally applied to the target population. Both deficiencies are related: an inability to predict progression in clinical trials will preclude the recruitment of populations at high risk of progression, necessary to increase the number of outcomes.

Prediction of CKD progression is based on the assessment of the estimated GFR (eGFR) and urinary albumin excretion (UAE)^1^. However, these parameters do not accurately predict CKD progression. eGFR only has a predictive value when the disease is advanced and 50% of renal function has already been lost, i.e. it is a late marker of disease progression. Moreover, many CKD patients in the G3a category^1,7^, the most prevalent CKD category, never progress, as recently observed for a classical albuminuric disease such as diabetic nephropathy. Although UAE is clinically useful to stratify risk, in some patients there are discrepancies between the urinary albumin creatinine ratio and 24h UAE^8^. Furthermore, recent reports have emphasized the risk of progressive CKD in normoalbuminuric individuals even for nephropathies classically associated with pathological albuminuria, such as diabetic kidney disease. In this regard, prediction of progression is especially challenging in patients with non-pathological UAE values and preserved (>60 ml/min/1.73 m^2^) eGFR, which represent the bulk of the general population and of high risk individuals. The Kidney Failure Risk Equation (KFRE) is a very recently developed prediction model based on demographical and laboratory parameters that has been shown to accurately predict progression to end-stage renal disease in patients with CKD stages 3–5^9,10^. The KFRE equation is shown in supplementary material.

An unmet medical need is the requirement of a set of biomarkers that improves the sensitivity of the current tools to predict CKD progression when eGFR is still >60 ml/min/1.73 m^2^, so that an early intervention can be initiated.

The urinary peptidome has been explored for novel biomarkers providing information on diagnosis and prognosis of CKD. A panel of 273 urinary peptides differentially expressed between healthy individuals and CKD patients defines the “CKD273 classifier”^11^. The diagnostic and prognostic value of the CKD273 classifier has been assessed in studies covering all stages of CKD^12–19^. The CKD273 classifier performed better than albuminuria in the early assessment of progressive renal disease^19^. The results of this study also indicated that molecular changes associated with CKD progression differ at different stages of CKD, and different molecular processes may be responsible for progression, depending on the stage of CKD. Based on this hypothesis, in the present study, we aimed to refine the urine peptidomics-based CKD273 classifier according to the baseline eGFR to improve prediction of the risk of rapid CKD progression. Patients were stratified according to baseline eGFR, and CKD273 subclassifiers were generated for each eGFR stratum. The performance of the CKD273 subclassifiers for rapid CKD progression prediction was compared with the current state of the art, albuminuria and KFRE. Additionally, they were compared with the original CKD273 classifier.

## Results

### Baseline demographical and clinical data

Table 1 shows the main demographical and clinical data from the 1482 patients included in the study, grouped into rapid progressors (N = 342) and non-rapid progressors (N = 1140). Rapid progression was defined according to international consensus criteria^1^. A majority of patients (74%) were diabetic and the proportion of diabetic patients was significantly higher among the rapid progressors (97% vs 68%, P < 0.001). Rapid progressors were older than non-rapid progressors (55.59 ± 12.49 vs 50.38 ± 15.16 years, P < 0.001) and had higher systolic blood pressure (133.69 ± 14.76 vs 127.96 ± 15.37 mmHg, P < 0.001), UAE (672.13 ± 1183.44 vs 106.23 ± 398.58 mg/24 h, P < 0.001) and CKD273 score (0.1619 ± 0.56 vs −0.289 ± 0.51, P < 0.001). By contrast, no differences were observed in diastolic blood pressure or baseline eGFR between both groups. A majority (71%) of patients did not fulfill the eGFR or UAE criteria to be diagnosed of CKD (eGFR was > 60 ml/min/1.73 m^2^ and UAE was < 30 mg/24 h). More than a half (56%) of patients classified as rapid progressors had eGFR > 60 ml/min/1.73 m^2^ and UAE < 30 mg/24 h. It should be noticed that a diagnosis of CKD entails a higher risk of CKD progression^1^. However, in the majority of rapidly progressing patients, CKD progression could not be predicted based on eGFR or UAE criteria.

**Table 1:** Demographical and clinical data of the study population.

Characteristic	All (n = 1482)	Rapid progressors (n = 342)	Non-rapid progressors (n = 1140)	P-value*
Women	669 (45.14)	149 (43.57)	520 (45.61)	<0.001
Diabetes mellitus	1104 (74.50)	331 (96.78)	773 (67.81)	<0.001
Age, years	51.59 ± 14.74	55.59 ± 12.49	50.38 ± 15.16	<0.001
Systolic pressure, mmHg	129.25 ± 15.42	133.69 ± 14.76	127.96 ± 15.37	<0.001
Diastolic pressure, mmHg	77.06 ± 8.77	76.38 ± 8.93	77.26 ± 8.72	0.29
eGFR (CKD-EPI), ml/min/1.73 m^2^	69.28 ± 19.50	66.04 ± 24.79	70.25 ± 17.50	0.46
eGFR < 60 ml/min/1.73 m^2^	361 (24.35)	124 (36.26)	237 (20.79)	<0.001
UAE, mg/24 h	236.82 ± 708.20	672.13 ± 1183.44	106.23 ± 398.58	<0.001
UAE > 30 mg/24 h	288 (19.43)	145 (42.40)	143 (12.54)	0.98
eGFR < 60 ml/min/1.73 m2 or UAE > 30 mg/24 h	430 (29.01)	152 (44.44)	278 (24.39)	<0.001
CKD273 score	−0.1848 ± 0.56	0.1619 ± 0.56	−0.2888 ± 0.51	<0.001
Follow-up, years	3.24 ± 1.18	2.48 ± 1.06	3.46 ± 1.12	<0.001
eGFR datapoints	4.54 ± 2.09	5.28 ± 1.97	4.32 ± 2.08	<0.001

Data expressed as n (%) or mean ± standard deviation. *P-values were calculated using Mann-Whitney test for continuous variables and ANOVA test for dichotomous variables. CKD273: urinary peptidomics classifier. UAE: urinary albumin excretion.

### CKD273 subclassifiers developed for each eGFR stratum

Since urinary peptidomics may change as kidneys are progressively more injured, reflecting structural kidney changes, the study population was distributed across different eGFR strata to optimize the CKD273 classifier based on relevant urinary peptides for each eGFR stratum (Table 2). The training and validation sets for each new CKD273 subclassifier are indicated in Table S1. In brief, CKD273 subclassifiers developed from data corresponding to one eGFR strata were validated in patients from different eGFR strata.

**Table 2:** Distribution of patients (N = 1482) in strata according to eGFR.

Stratum	Baseline eGFR (ml/min/1.73 m^2^)	Rapid progressors	Non-rapid progressors	Total
1	≥80	129	350	479
2	70–79	64	317	381
3	60–69	25	236	261
4	50–59	16	110	126
5	40–49	26	41	67
6	30–39	44	36	80
7	<30	38	50	88

**Figure 1:**
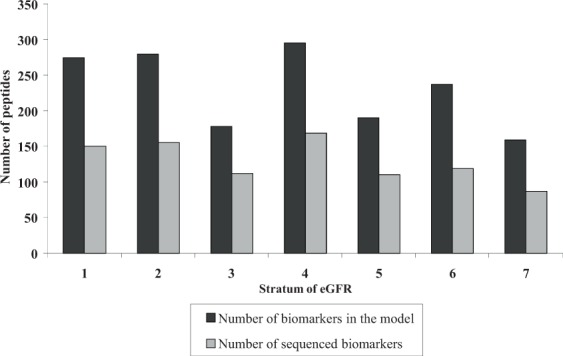
Distribution of the peptides in the CKD273 subclassifiers developed for each eGFR stratum. Black bars represent the total number of peptide biomarkers in the CKD273 subclassifiers. The number of sequenced biomarkers is represented by grey bars.

Figure 1 shows the different number of peptides used for the CKD273 subclassifiers generated for each eGFR stratum. The number of peptides in the CKD273 subclassifiers was variable, oscillating between 159 in the subclassifier developed from stratum 7 to 296 peptides in the subclassifier developed from stratum 4. Both sequenced and non-sequenced peptides were included in the CKD273 subclassifiers. Sequenced peptides represented approximately half of the total number of peptides in the subclassifiers.

Table 3 shows AUCs for predicting rapid CKD progression for the CKD273 subclassifiers obtained in the training sets, as well as the CKD273 subclassifiers with higher AUC in the validation sets for each eGFR stratum. Overall, the performance of the CKD273 subclassifiers to predict rapid progression was better in strata with closer baseline levels of eGFR in the validation and training sets. As an example, for patients with baseline eGFR ≥ 80 ml/min/1.73 m^2^, the classifier with best performance in the validation set had been generated in the 60–69 ml/min/1.73 m^2^ training set (Table 3). Table S2 presents the AUCs for prediction of rapid progression obtained in the validation sets of each CKD273 subclassifier. Table S3 shows CKD273 subclassifier scores for rapid and non-rapid progressors for the best performing CKD273 subclassifiers in the validation sets in each stratum of baseline eGFR. The frequency graphs for CKD273 subclassifiers in each stratum, along with their cut-off values, are depicted in Supplementary Fig. 1, while the sensitivity, specificity, and positive and negative predictive values of the best performing CKD273 subclassifiers in each stratum are presented in Supplementary Fig. 2.

**Table 3:** Prediction of rapid CKD progression.

Baseline eGFR stratum (eGFR)	AUC (95% CI) in the CKD273 subclassifier generated in the training set	Best performing CKD273 subclassifier in this eGFR stratum (validation set)**	AUC (95% CI) for best performing CKD273 subclassifier in this eGFR stratum (validation set)	AUC (95% CI) for albuminuria	AUC (95% CI) for CKD273	KFRE AUC (95% CI) for ESRD at 2 years	KFRE AUC (95% CI) for ESRD at 5 years
1 (≥80)	0.920 (0.892–0.943)	60–69	0.741 (0.683–0.793)	0.636 (0.591–0.679)	0.719 (0.677–0.759)	0.514 (0.468–0.559)	0.604 (0.559–0.648)
2 (70–79)	0.810 (0.767–0.848)	60–69	0.797 (0.743–0.844)	0.568 (0.517–0.619)	0.714 (0.666–0.759)	0.557 (0.505–0.607)	0.610 (0.559–0.659)
3 (60–69)	0.842 (0.792–0.884)	≥80	0.778 (0.738–0.815)	0.685 (0.624–0.740)	0.684 (0.624–0.740)	0.524 (0.462–0.586)*	0.511 (0.449–0.573)*
4 (50–59)	1.000 (0.971–1.000)	40–49	0.824 (0.712–0.906)	0.773 (0.690–0.843)	0.751 (0.666–0.823)	0.712 (0.624–0.789)	0.700 (0.612–0.778)
5 (40–49)	0.958 (0.879–0.992)	50–59	0.728 (0.741–0.803)	0.871 (0.766–0.940)	0.726 (0.603–0.828)	0.824 (0.711–0.906)	0.824 (0.711–0.906)
6 (30–39)	0.985 (0.929–0.999)	40–49	0.728 (0.605–0.830)	0.789 (0.683–0.872)	0.623 (0.508–0.729)	0.688 (0.574–0.787)	0.688 (0.574–0.787)
7 (<30)	0.949 (0.880–0.985)	30–39	0.690 (0.577–0.789)	0.728 (0.623–0.818)	0.534 (0.425–0.641)*	0.591 (0.481–0.694)*	0.591 (0.481–0.695)*

Area under the curve (AUC) and 95% confidence interval (95% CI) of the performance of the CKD273 subclassifiers in the training and in the best validation set. Performances of albuminuria and the CKD273 classifier are also displayed. eGFR expressed as ml/min/1.73 m^2^. The Kidney Failure Risk Equation (KFRE) allows estimating the risk of progression to end-stage renal disease (ESRD) within a fixed temporal horizon. ^*^P-values were non-significant.

**Number indicates in which training set was the CKD273 subclassifier generated that performed best in the validation set for the stratum shown in column 1.

### Comparison of the CKD273 subclassifiers with albuminuria and the CKD273 classifier to predict rapid CKD progression

**Figure 2:**
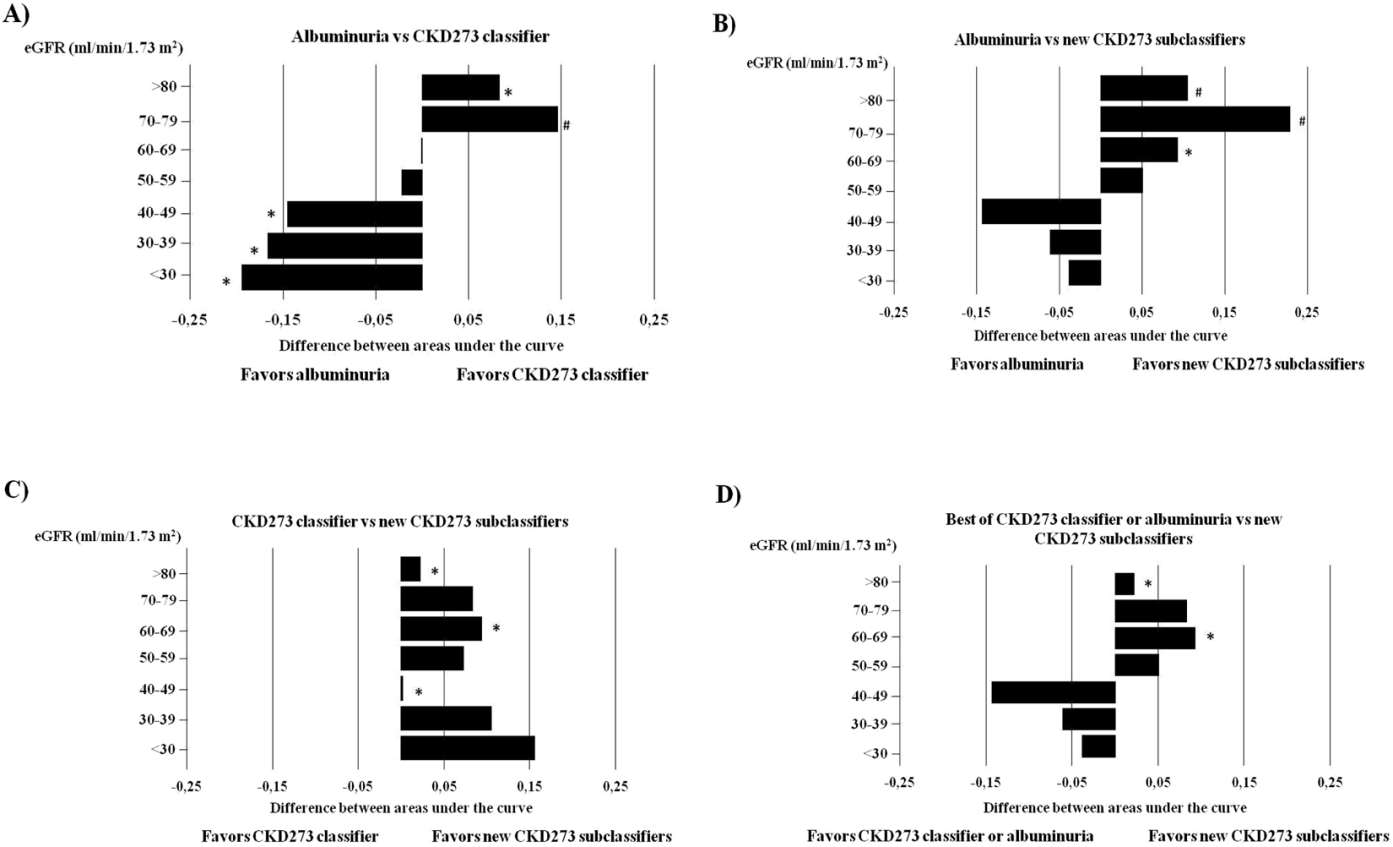
Comparative performances of predictors of CKD progression. (**A**) When comparing the performances of albuminuria and the CKD273 classifier in each eGFR stratum, CKD273 classifier outperformed albuminuria in patients with eGFR > 70 ml/min/1.73 m^2^. (**B**) The CKD273 subclassifiers performed better in predicting rapid CKD progression in the strata of patients with eGFR > 60 ml/min/1.73 m^2^. (**C**) The CKD273 subclassifiers developed for specific ranges of eGFR were statistically superior to CKD273 classifier in strata 1 and 3. (**D**) The performance of the best of either CKD273 classifier (strata corresponding to an eGFR > 70 ml/min/1.73 m^2^) or albuminuria (eGFR < 70 ml/min/1.73 m^2^) in each stratum was compared with that of the CKD273 subclassifiers, finding an improvement of the latter in strata of patients with eGFR > 60 ml/min/1.73 m^2^, that reached statistical significance in patients with eGFR > 80 ml/min/1.73 m^2^. The horizontal axis displays the difference between the AUC of both comparators for each figure. ^*^P < 0.05 and ^#^P < 0.001 vs the contrasted variable.

The performances of albuminuria and CKD273 classifier for predicting rapid CKD progression are shown in Table 3. The AUC for albuminuria was superior in strata with baseline eGFR ≤ 50 ml/min/1.73 m^2^ (strata 5, 6 and 7), while the AUC for CKD273 classifier was significantly higher than the AUC for albuminuria if baseline eGFR was ≥70 ml/min/1.73 m^2^ (strata 1 and 2, Fig. 2A). No differences between albuminuria and CKD273 classifier were observed in strata 3 and 4.

We next compared the CKD273 subclassifiers versus albuminuria to determine whether they improved the prediction of rapid CKD progression. The CKD273 subclassifiers performed better in the validation set than albuminuria in early stages of CKD (eGFR ≥ 60 ml/min/1.73 m^2^, Table 3 and Fig. 2B). Particularly, the AUC obtained in the stratum 1 (highest eGFR) validation cohort for the CKD273 subclassifiers generated in strata 2 and 3 was significantly higher than the AUC of albuminuria (0.782, 95% CI 0.743–0.819, P < 0.001 and 0.778, 95% CI 0.738–0.815, P < 0.001, respectively) (Table 3, Fig. 2B, Table S2). A similar improvement was observed when the CKD273 subclassifiers developed for strata 1 and 3 were validated in stratum 2 (0.736, 95% CI 0.689–0.780, P < 0.001 and 0.751, 95% CI 0.705–0.794, P < 0.001, respectively).

Next, the CKD273 subclassifiers were compared with the CKD273 classifier (Fig. 2C). The performance to predict rapid CKD progression in the validation set of the CKD273 subclassifiers developed in patients with preserved eGFR (eGFR ≥ 60 ml/min/1.73 m^2^) was superior to that of CKD273 classifier. Especially, the AUC for the validation in eGFR stratum 1 of the CKD273 subclassifiers generated for strata 2 and 3 were higher than those of the CKD273 classifier (0.782, 95% CI 0.743–0.819, P < 0.01 and 0.778, 95% CI 0.738–0.815, P < 0.01, respectively) (Table S2). In addition, the AUC in the validation in strata 3 and 5 of the CKD273 subclassifiers developed in strata 2 and 4, respectively, also performed better than the CKD273 classifier (0.797, 95% CI 0.743–0.844, P < 0.05 and 0.824, 95% CI 0.712–0.906, P < 0.05, respectively).

Additionally, in the validation set, the CKD273 subclassifiers were superior to the best of either albuminuria or CKD273 classifier to predict rapid CKD progression for strata with baseline eGFR ≥ 60 ml/min/1.73 m^2^ (Fig. 2D).

### Comparison of the CKD273 subclassifiers with the Kidney Failure Risk Equation to predict rapid CKD progression

We applied the 4-variable KFRE to calculate the estimated risk of progression to end-stage renal disease at 2 and 5 years. AUCs obtained from the KFRE at both time points studied are displayed in Table 3. Table S4 shows the percentage risk of renal failure at 2 and 5 years in rapid progressors and non-rapid progressors in each stratum of eGFR. Supplementary Figs 3 and 4 reflect the frequency graphs for the risk of renal failure at 2 and 5 years in rapid progressors and non-rapid progressors, calculated for each stratum by applying KFRE. In addition, the sensitivity, specificity, and positive and negative predictive values of the risk of kidney failure calculated from KFRE are depicted in Supplementary Figs 5 and 6.

The best performances of KFRE at 2 and 5 years were found in patients with eGFR between 40 and 59 ml/min/1.73 m^2^. For patients with eGFR 40–49 ml/min/1.73 m^2^ the AUC at 2 and 5 years was 0.824, 95% CI 0.711–0.906, whereas in patients with 50–59 ml/min/1.73 m^2^ the AUCs at 2 and 5 years were 0.712, 95% CI 0.624–0.789 and 0.700, 95% CI 0.612–0.778, respectively (Table 3).

The performance of the CKD273 subclassifiers was significantly better than those of the KFRE equation at 2 and 5 years for patients with eGFR ≥ 80 ml/min/1.73 m^2^ (P < 0.0001 at both 2 and 5 years), 70–79 ml/min/1.73 m^2^ (P = 0.0002 and P = 0.0165 at 2 and 5 years, respectively) and 60–69 ml/min/1.73 m^2^ (P = 0.0207 and P = 0.0077 at 2 and 5 years, respectively). That is, the CKD273 subclassifiers outperformed KFRE for patients not meeting the current thresholds to diagnose CKD based on baseline eGFR values. For eGFR < 50 ml/min/1.73 m^2^, CKD273 subclassifiers exhibited a statistically similar performance to KFRE at both time points assessed.

### Prediction of rapid eGFR loss in patients without CKD

In patients with non-pathological albuminuria there is currently no biomarker that can predict eGFR loss before CKD is diagnosed, or in other words, before eGFR falls below 60 ml/min/1.73 m^2^. Thus, we assessed the performance of the urinary peptidomics CKD273 subclassifiers to predict eGFR loss in patients without CKD (eGFR ≥ 60 ml/min/1.73 m^2^ and UAE < 30 mg/24 h). In these individuals, who would be considered to have healthy kidneys according to the current KDIGO definitions of CKD, the CKD273 subclassifiers predicted a rapid loss of eGFR with AUC that ranged from 0.797 (0.743–0.844) for the stratum 3 validation cohort to 0.736 (0.689–0.780) for the stratum 2 validation cohort (Table S5).

### Risk of rapid CKD progression associated with the CKD273 subclassifiers in early CKD or without CKD

Cox analysis disclosed that the adjusted hazard ratios (95% CI) of rapid CKD progression associated with the new validated CKD273 subclassifiers were 1.91 (1.56 to 2.35), 2.19 (1.62 to 2.96), and 4.57 (2.17 to 9.59) for patients with eGFR ≥ 80, 70–79, and 60–69 ml/min/1.73 m^2^, respectively, when adjusted for albuminuria, DM, and baseline eGFR (Table S6). When only subjects with UAE < 30 mg/24 h were considered, the hazard ratios (95% CI) of rapid CKD progression associated with the new CKD273 subclassifiers were 1.83 (1.49 to 2.24), 1.97 (1.44 to 2.68), and 4.08 (1.88 to 8.83) for patients with eGFR ≥ 80, 70–79, and 60–69 ml/min/1.73 m^2^, respectively (Table S7).

In a sensitivity analysis, similar results were obtained when assessing the association between the new validated CKD273 subclassifiers and rapid progression in models adjusted for sex, age, and systolic blood pressure in addition to albuminuria, DM, and baseline eGFR (Tables S6 and S7). The new CKD273 subclassifiers outperformed the original CKD273 classifier for patients in strata 1 and 3.

### Risk of CKD progression associated with the new classifiers in early CKD or no CKD in diabetics

Since the vast majority of rapid progressors were diabetics and given that diabetes is an effect modifier in the non-rapid progressor subpopulation in the majority of eGFR strata assessed (Table S8), we also evaluated the risk of rapid CKD progression associated with the new CKD273 subclassifiers in an analysis restricted to diabetic patients.

In a multivariate model adjusted by albuminuria and baseline eGFR in individuals with baseline preserved eGFR (>60 ml/min/1.73 m^2^), the hazard ratios (95% CI) of CKD progression associated with the CKD273 subclassifiers were 1.86 (1.52 to 2.38), 2.20 (1.60 to 3.02), and 5.09 (2.30 to 11.31) for patients with eGFR ≥ 80, 70–79, and 60–69 ml/min/1.73 m^2^ (Table S9). When the analysis was restricted to patients with normal renal function and UAE < 30 mg/24 h, the hazard ratios (95% CI) of rapid CKD progression associated with the CKD273 subclassifiers were 1.86 (1.52 to 2.29), 2.08 (1.50 to 2.88), and 4.64 (2.03 to 10.59) (Table S10).

The sensitivity analyses disclosed similar results when the models were adjusted for sex, age, and systolic blood pressure in addition to albuminuria and baseline eGFR (Tables S9 and S10).

### Analysis of the peptides most differentially expressed in each CKD273 subclassifier

The identification of urinary peptides may help trace the pathophysiological changes that mediate CKD progression. We therefore examined the most significant differentially expressed peptides between rapid progressors and non-rapid progressors in each stratum of eGFR. To avoid bias due to the different number of subjects included in each group, only the ten most differentially expressed peptides were investigated (Table 4, supplementary material, Table S11).

**Table 4:** Distribution of the 10 most significant sequenced peptides differentially expressed between rapid progressors and non-rapid progressors in each baseline eGFR stratum.

Stratum	Protein name	Number of peptides	Number of peptides overlapping with the CKD273 classifier
1	Collagen alpha-1(I) chain	7	5
	Uromodulin	2	1
	Sodium/potassium-transporting ATPase subunit gamma	1	1
2	Collagen alpha-1(I) chain	6	5
	Collagen alpha-2(I) chain	2	1
	Collagen alpha-1(II) chain	1	—
	Sodium/potassium-transporting ATPase subunit gamma	1	—
3	Collagen alpha-1(I) chain	7	4
	Collagen alpha-1(III) chain	1	—
	Collagen alpha-1(XIV) chain	1	—
	Collagen alpha-2(I) chain	1	1
4	Alpha-1-antitrypsin	4	2
	Alpha-1B-glycoprotein	1	—
	Apolipoprotein A-IV	1	—
	Complement C3	1	—
	Cornulin	1	—
	Retinol-binding protein 4	1	—
	Serum paraoxonase/arylesterase 1	1	—
5	Collagen alpha-1(I) chain	5	4
	Collagen alpha-1(II) chain	1	—
	Collagen alpha-1(XIV) chain	1	—
	Collagen alpha-2(IX) chain	1	—
	Fibrinogen alpha chain	1	1
	Serum albumin	1	1
6	Beta-2-microglobulin form pI 5.3	2	—
	Antithrombin-III	1	—
	Apolipoprotein A-IV	1	—
	Collagen alpha-1(II) chain	1	—
	Collagen alpha-1(XIX) chain	1	—
	Complement C3	1	—
	Peptidase inhibitor 16	1	—
	Apolipoprotein A-I	1	—
	Unconventional myosin-Ib	1	—
7	Collagen alpha-1(I) chain	3	1
	Collagen alpha-1(III) chain	2	1
	Collagen alpha-1(XVI) chain	1	—
	Collagen alpha-2(V) chain	1	—
	Protein S100A9 (Calgranulin B)	1	—
	Serum amyloid A-2 protein	1	—
	T-lymphoma invasion and metastasis-inducing protein 1	1	—

**Figure 3:**
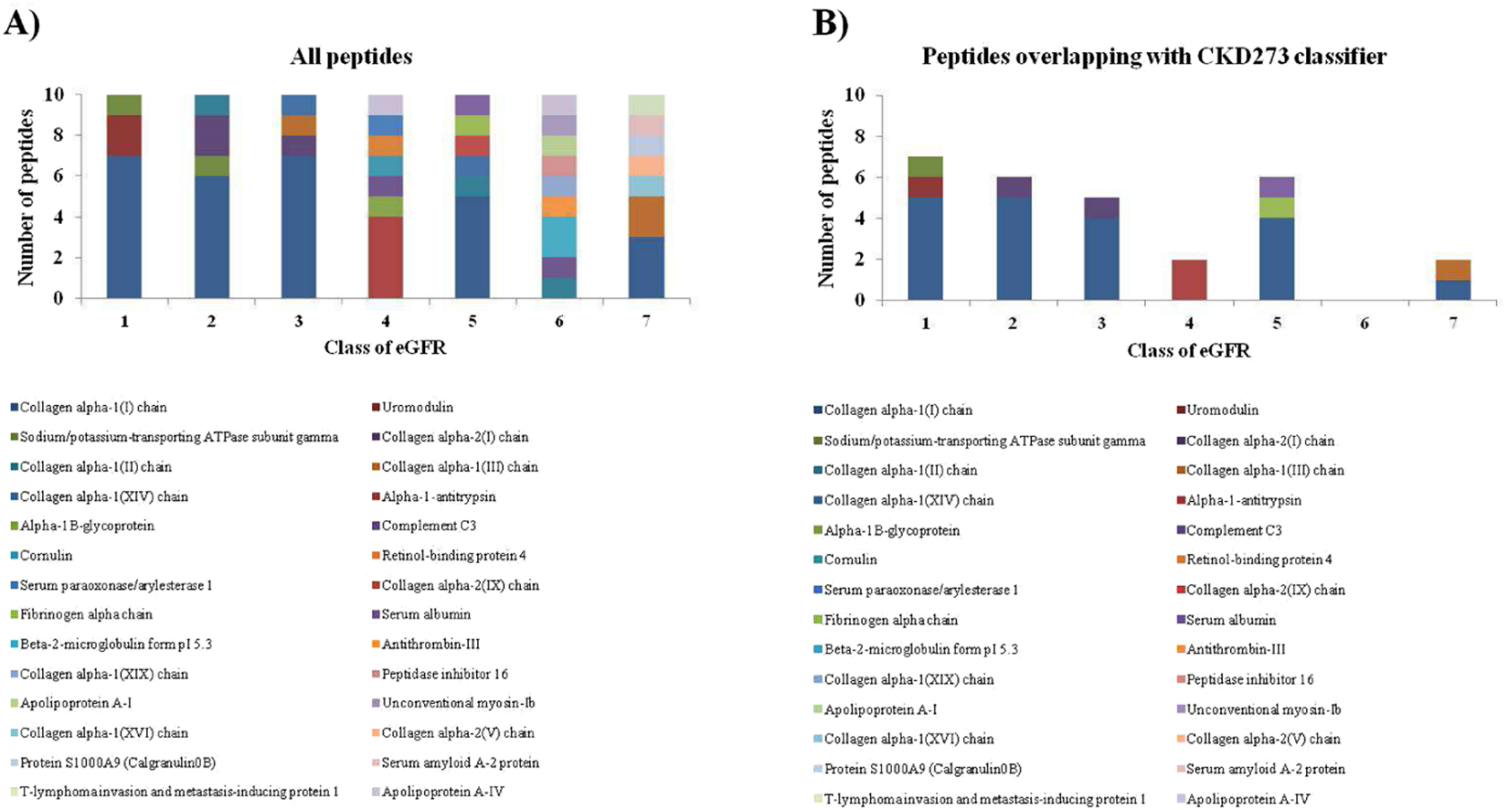
Composition of the CKD273 subclassifiers. (**A**) Number and protein origin of the ten most differentially expressed peptides between rapid progressors and non-rapid progressors in each eGFR stratum. (**B**) Number of peptides overlapping with the CKD273 classifier in each eGFR stratum.

In patients with eGFR ≥ 60 ml/min/1.73 m^2^, differentially expressed peptides between rapid progressors and non-rapid progressors were predominantly collagen fragments that were down-regulated in rapid progressors (Fig. 3A). In more advanced CKD (eGFR ≤ 50 ml/min/1.73 m^2^), peptides belonged to a different pattern of proteins (Fig. 3A). When the peptide overlap with CKD273 was investigated, the highest number of common peptides was observed in stratum 1, whereas in stratum 6 there were no peptides overlapping with the CKD273 classifier (Table 4, Fig. 3B).

## Discussion

The present study was designed to define biomarker CKD273 subclassifiers for different degrees of CKD that improve prediction of CKD progression over the currently available albuminuria and CKD273 classifier. This may allow an early identification of the risk of rapid progressive loss of eGFR even before CKD can be diagnosed by current standards^13,15,17–19^. The newly generated CKD273 subclassifiers were developed in cohorts covering all stages of CKD and were validated in a different eGFR stage close to that in which the classifiers were developed. This design was chosen based on the assumption that the specific cut-offs for the different strata are arbitrary (as eGFR is a continuous variable) and neighboring strata should be of substantial similarity with respect to molecular pathophysiology, and to preserve as much power as possible by employing the complete stratum for discovery and subclassifier development. The main finding is that the newly developed urinary peptidomics CKD273 subclassifiers outperform both albuminuria and the CKD273 classifier for the prediction of rapid CKD progression in early CKD (>60 ml/min/1.73 m^2^) and even predict a rapid loss of eGFR in individuals who do not meet current criteria of CKD diagnosis, i.e. those with eGFR ≥ 60 ml/min/1.73 m^2^ and UAE < 30 mg/24 h, representing the bulk of the general population.

The diagnosis and categorization of CKD is based on the estimation of eGFR from serum creatinine, together with the determination of UAE^20^. A diagnosis of CKD is associated with an increased risk of CKD progression^1^. However, these parameters may not result accurate enough to predict CKD progression at early CKD stages^21,22^. An unmet clinical need is for biomarkers that increase the predictive accuracy for CKD progression in patients at early CKD stages or in patients at high risk of CKD, such as diabetics who do not yet fulfill the criteria to diagnose CKD. Biomarkers should predict progression before eGFR decreases below the threshold of established CKD or albuminuria becomes pathological. This may allow prompt therapeutic intervention thus increasing the chance of success^23^. CE-MS has provided novel insights into renal or urological diseases^24–29^. In CKD, the analysis of urinary peptides enabled the generation of the CKD273 classifier, a SVM model constituted by 273 peptides that differentiates between healthy and CKD subjects^11^.

Combination of a high number of peptides as SVM models frequently outperforms individual biomarkers for diagnostic and prognostic purposes. We have now developed novel SVM CKD273 subclassifiers based on urine peptidomics, one for each eGFR stratum, and validated them in the remaining strata of patients. The new CKD273 subclassifiers could only be validated in those eGFR strata close to the eGFR strata where they were developed. Interestingly, the CKD273 subclassifier developed in baseline eGFR stratum 4 (eGFR 50–59 ml/min/1.73 m^2^) displayed a very high AUC [1.000 (0.971–1.000)] in the training set, that was not matched by validating in stratum 4 the CKD273 subclassifiers generated in adjacent strata, although the AUC for the validated subclassifiers was still high [0.824 (0.712–0.906)]. While this may represent overfitting in the discovery set, it is worth exploring the validation of this specific stratum 4 CKD273 subclassifier in additional cohorts, given the critical importance of this eGFR range, currently considered diagnostic of CKD if persistent for 3 months or longer, but with a high proportion of patients never progressing to more advance stages.

UAE is used to diagnose and monitor renal disease. However, albumin excretion is highly variable within individuals^8^ and, more importantly, the prevalence of pathological albuminuria increases with decreasing eGFR^30^. The results presented here are in line with this observation, since the performance of albuminuria for predicting CKD progression was higher in strata with lower eGFR. In this regard, albuminuria outperformed the CKD273 subclassifiers in predicting CKD progression in the validation set in patients who have a eGFR which diagnosis of CKD (<60 ml/min/1.73 m^2^). However, this population is already known to have a high risk of progression based on the baseline eGFR. The new CKD273 subclassifiers are superior to albuminuria in predicting CKD progression in patients with eGFR ≥ 60 ml/min/1.73 m^2^. This is an especially relevant population: it is the most frequent eGFR category in the general population and given the normal eGFR, the diagnosis of CKD is usually based on the presence of pathological albuminuria. Thus, the finding of a lab criterion that outperforms albuminuria is predicting CKD progression in this population, which in the absence of albuminuria would not be diagnosed of CKD, is especially relevant. Parameters that provide prognostic information when albuminuria is within physiological range may allow an early diagnosis of CKD in this population. Additionally, the new CKD273 subclassifiers outperformed the CKD273 classifier in CKD progression in all eGFR strata. Several studies have confirmed the value of the CKD273 classifier to predict worsening of albuminuria^13,15^, as well as progression of CKD^14,17–19,31^. We have also assessed the risk of our population to progress to end-stage renal disease based on the calculation of the 4-variable KFRE. The best performance of this predictive model in our patients was observed in strata of patients with eGFR 40–50 ml/min/1.73 m^2^, but the CKD273 subclassifiers outperformed KFRE when eGFR ≥ 60 ml/min/1.73 m^2^ were significantly superior to those of KFRE at both 2 and 5 years. This is in agreement with the current literature since the predictive value of KFRE has been shown in CKD stages 3–5^9,10^. Taken together, these findings reveal an improvement over the current approaches for predicting rapid CKD progression in CKD patients and non-CKD individuals with eGFR > 60 ml/min/1.73 m^2^. In this regard, 13% of patients who progressed did so despite normal albuminuria and eGFR. The current definition of CKD is based on the health implications of abnormalities of kidney structure or function persisting for more than 3 months^1^. One of these adverse consequences for health is the progressive loss of renal function. In this context, the magnitude of the risk of eGFR progression associated to the new urine peptidomics CKD273 subclassifiers in adjusted models (HR 2.2 to 5.2 for patients with eGFR > 60 ml/min/1.73 m^2^) is similar or greater than that associated with albuminuria category A2 as per the KDIGO review of data^1^. Thus, if the data presented here are validated in larger, independent cohorts, it is conceivable that in the future an abnormal urine peptidomics pattern will be incorporated into an updated definition of early stage CKD.

The distinctive pattern of peptides found between rapid progressors and non-rapid progressors was notably different in the higher and lower eGFR strata. The CKD273 subclassifiers developed in strata with eGFR ≥ 60 ml/min/1.73 m^2^ were abundant in collagen fragments, suggesting structural extracellular matrix changes^32,33^. Lower amounts of specific uromodulin peptides were found in rapid progressors, which may represent lower tubular mass. In more advanced stages of CKD (eGFR < 49 ml/min/1.73 m^2^), peptides derived from plasma proteins were abundant and associated to faster progression, maybe indicating more severe glomerular injury^19^.

We also investigated the overlap between the CKD273 classifier and the CKD273 subclassifiers developed for each eGFR stratum. The number of common peptides between the classifiers was variable according to eGFR strata. The CKD273 classifier was generated from a large cohort of patients in different CKD stages^11^. As abovementioned, the pattern of urinary peptides may change substantially as CKD progresses. Therefore, it is not unexpected that there is partial overlap between the CKD273 classifier and the CKD273 subclassifiers. The variability of the urinary peptidome across CKD categories may also explain the better performance of eGFR category-specific subclassifiers over the CKD273 classifier.

Several weaknesses of the present report should be acknowledged. Firstly, eGFR, and not measured GFR was used to assess CKD. However, as eGFR is a well-accepted parameter and measurement of GFR is quite demanding, this compromise appears appropriate. To increase certainty on the assessment (rapid progressor or non-rapid progressor), patients in a “grey zone” (loss of eGFR between 1.5 and 5 ml/min/1.73 m^2^/year) were not included in the discovery study. This approach further reduced inaccuracy of eGFR. A majority of patients were diabetic. Thus, the CKD273 subclassifiers may not perform equally well in cohorts where a majority of patients are not diabetic or have lower risk of CKD progression. However, diabetes is the most common cause of end-stage renal disease. In this regard, an unmet clinical need is the prediction of rapid eGFR loss in diabetics without pathological albuminuria^34^. The availability of several subclassifiers for patients with eGFR ≥ 60 ml/min/1.73 m^2^ according to eGFR strata may be confusing for the clinician and the development of a single classifier with a clear cut-off point for these patients would be desirable. The ideal outcome measure would have been end-stage kidney disease. However, the long natural history of most CKD causes makes it unfeasible to use as a single primary outcome measure in clinical studies. Most AUCs are around 0.8, falling short of diagnostic test standards, although they may be useful to enrich clinical trials in patients at high risk of progression. The study comprised several independently generated cohorts from four continents and discovery and validation subcohorts differed for each biomarker, but still, no external validation cohort is available. Finally, information on the use of renin angiotensin system blockers was not available for a large number of patients, precluding analysis of this variable.

In summary, we now present refined urinary biomarker CKD273 subclassifiers developed and validated for different strata of baseline eGFR. These CKD273 subclassifiers performed better than albuminuria or the CKD273 classifier at predicting rapid loss of eGFR, particularly in patients with early CKD, with better preserved renal function (eGFR ≥ 60 ml/min/1.73 m^2^) and even in individuals who did not fulfill the eGFR and albuminuria diagnosis thresholds for a diagnosis of CKD. Improved identification of patients at high risk of rapid progression at early CKD stages may be useful to enrich the clinical trials of early intervention in high-risk individuals. Confirmation of these findings in a larger cohort of patients may serve as the basis to expand the current concept of CKD to also encompass the presence of a pathological urinary peptidomics pattern. This would allow an earlier diagnosis of CKD, thus increasing the chances of successful therapeutic intervention.

## Methods

### Patient data

Patient data were collected from the Human Urinary Proteome Database, which contains data from different independent cohorts as explained below^19,35–37^. CKD had been categorized according to local clinical care and the best adherence to international guidelines. Specific CKD diagnoses were biopsy-proven for glomerulonephritides, while diabetic nephropathy was diagnosed clinically when diabetic patients showed clinical parameters (urinary albumin, eGFR) consistent with kidney disease during follow-up in the absence of evidence for a different nephropathy. Out of 2422 patients from previous studies with available baseline and follow-up eGFR^13,17,18,38–43^, 1482 met the definitions of either rapid progressor (sustained decline in eGFR of 5 ml/min/1.73 m^2^/year or more) or non-rapid progressor/stable renal function (change between −1.5 and +5 ml/min/1.73 m^2^/year), as detailed below. The choice of the 5 ml/min/1.73 m^2^/year was not arbitrary. This is the definition of rapid progression according to the consensus international KDIGO guidelines^1^. The number of participants in the Human Urine Proteome Database complying with the aforementioned eligibility criteria totaled 1482. The patient cohorts have been described in greater detail in prior studies and participants were: (i) patients enrolled in the Diabetes Retinopathy Candesartan Trials in type 1 (DIRECT1)^44^ and type 2 (DIRECT2)^45^ diabetic patients; (ii) type 2 diabetic patients recruited into a Dutch study (PREDICTIONS)^39^, aiming at identifying disease-pathway specific biomarkers; (iii) diabetic patients recruited at clinics in Australia^46^ and Hannover, Germany^47^, and individuals in the Flemish Study on Environment, Genes and Health Outcomes (FLEMENGHO)^17^. Demographic and clinical data of the study population are summarized in Table 1.

The definition of CKD stages was based on eGFR using the Chronic Kidney Disease in Epidemiology Collaboration (CKD-EPI) equation^48^. The majority (>90%) of samples were obtained from European sources and no information on race or ethnicity was available on most patients. The proportion of patients on renin-angiotensin system blockade ranged from 45 to 95% within the different strata, but this information was not collected from all the patients included in the study.

The studies were conducted according to the regulations on the protection of individuals participating in medical research and conform to the principles of the Declaration of Helsinki and informed consent was provided by all patients participating in the aforementioned studies. All datasets were received anonymized. Studies from which the data were extracted had been approved before their launch by the Hanover Medical School Ethics Committee (No. 3116–2016).

### CE-MS data and CKD273 classifier analysis

Sample collection and analysis of urine samples using capillary electrophoresis-mass spectrometry (CE-MS) have been previously reported^17,18,41–43^. Normalized signal intensity was used as a measure for relative abundance for peptide quantification. For normalization of analytical and urine dilution variances, MS signal intensities were normalized relative to 29 housekeeping peptides with small relative standard^49^. The mean peptide relative abundance was calculated by multiplying the mean of the amplitudes of each individual peptide with its frequency, defined by the occurrence of each peptide present in the cohort. Fold change of urinary peptides was calculated as amplitude of urinary peptides in rapid progressors (cases) divided by the amplitude of urinary peptides in non-rapid progressors (controls). The CKD273 classifier is a support vector machine (SVM) classification model^50,51^ based on the amplitudes of 273 urinary peptides^11^, which allows the calculation of a classification score for the presence and progression of CKD.

### Generation of CKD273 subclassifiers

Patients were stratified into seven eGFR strata based on baseline eGFR values (Table 2). The normalized levels of urinary peptides were compared between rapid progressors and non-rapid progressors (see below) for the identification of potential biomarkers associated to risk of progression of CKD in each stratum of eGFR. We identified peptides differentially expressed between rapid progressors and non-rapid progressors of the specific eGFR stratum with the multivariate statistical analysis. The significant peptides for each eGFR stratum were combined with the 273 peptides of the CKD273 classifier and were included in specific SVM models^52^. The SVM model (type C-SV) was generated with the cran R-package e01071^53^ using Gaussian basis radial functions as the kernel. The SVM models were optimized by the take-one-out procedure (systematic reduction of peptides in the classifier) with respect to an optimal result after cross-validation, the consistent regulation and a frequency over 30% of the relevant peptides.

### Training and validation sets

The training sets for the CKD273 subclassifiers were the specific eGFR strata where the subclassifiers were generated, whereas the validation sets were the remaining eGFR strata that were not used to generate the specific CKD273 subclassifier. Thus, for example, the CKD273 subclassifier generated in eGFR stratum 1 (patients with baseline eGFR ≥ 80 ml/min/1.73 m^2^) was independently validated in patients from each of the other different 6 baseline eGFR strata (Table S1). The best validation set was considered to be the baseline eGFR stratum where the CKD273 subclassifier developed for a different baseline eGFR stratum performed the best to predict rapid CKD progression (Table S2).

### Definition of rapid progression

The available eGFR values (average of five measurements with a minimum of two) over 39.1 ± 13.4 months were used to calculate the slope of eGFR per year. According to Kidney Disease Improving Global Outcomes (KDIGO) 2012 guidelines, rapid CKD progression was defined as a sustained decline in eGFR of 5 ml/min/1.73 m^2^ per year or more^1,54^. Thus, rapid progressors (cases) were patients with a decline in eGFR of >5 ml/min/1.73 m^2^/year, and were compared with non-rapid progressors (controls), with a slope between −1.5 and +5 ml/min/1.73 m^2^/year. Patients with a decline in eGFR between 5 and 1.49 ml/min/1.73 m^2^/year (eGFR slope −1.49 to −5 ml/min/1.73 m^2^/year), and those with an apparent increase of >5 ml/min/1.73 m^2^/year (eGFR slope >+5 ml/min/1.73 m^2^/year) were excluded from the analysis.

### Calculation of the estimated risk of progression to end-stage renal disease

The Kidney Failure Risk Equation is a model aimed to predict progression to end-stage renal disease on the basis of demographical and laboratory parameters^9^. The estimated risk of progression was calculated according to four parameters: sex, age, eGFR, and urinary albumin to creatinine ratio. We used the equation to estimate the risk of progression to end-stage renal disease within 2 and within 5 years. Given the complexity of the KFRE equation and for practical purposes, an app provided in the paper by Tangri *et al*.^9^ was used to calculate the risk of renal failure. This tool can be found at: https://qxmd.com/calculate/calculator_308/kidney-failure-risk-equation-4-variable.

### Statistical analysis

In order to develop biomarker CKD273 subclassifiers in patients with similar clinical characteristics, the study population was stratified according to the baseline eGFR determined by the CKD-EPI equation^48^ into seven strata: eGFR of >80, 70–79, 60–69, 50–59, 40–49, 30–39, and <30 ml/min/1.73 m^2^, respectively. The areas under the curve (AUC) for prediction of rapid eGFR loss by the CKD273 subclassifiers generated for each stratum, by the CKD273 classifier and by albuminuria were calculated by using ROC analysis with MedCalc software (version 12.1.0.0, MedCalc Software, Mariakerke, Belgium). We applied Cox proportional-hazards regression analysis to calculate the hazard ratios of CKD progression associated with the new validated classifiers. The remaining variables of interest with which models were built are described along with the results obtained, the time to event corresponded to the follow-up period for each patient, and the endpoint was their diagnosis as rapid progressor. As peptide profiles across the samples were not normally distributed, we used the non-parametric Wilcoxon rank sum test to calculate the P-values of individual peptides between rapid progressors and non-rapid progressors. Wilcoxon-test is best suited for the definition of potential biomarkers. Adjustment for multiple testing is needed to enable definition of reliable biomarkers^55^. Thus, the false discovery rate adjustments of Benjamini-Hochberg were used to correct for multiple testing^56^. The analysis was performed using proprietary software (R-based statistic software, version 2.15.3).

## Electronic supplementary material

Supplementary information

## Data Availability

Data generated or analysed during this study are included in this published article along with its Supplementary Information file. Additional data will be available from the corresponding author on reasonable request.
