# Publisher Correction: Novel Urinary Biomarkers For Improved Prediction Of Progressive eGFR Loss In Early Chronic Kidney Disease Stages And In High Risk Individuals Without Chronic Kidney Disease

**DOI:** 10.1038/s41598-018-36919-7

**Published:** 2018-12-10

**Authors:** María E. Rodríguez-Ortiz, Claudia Pontillo, Mariano Rodríguez, Petra Zürbig, Harald Mischak, Alberto Ortiz

**Affiliations:** 1 Instituto de Investigación Sanitaria Fundación Jiménez Díaz, Fundación Renal Iñigo Álvarez de Toledo, Universidad Autónoma de Madrid. REDinREN, Avda. Reyes Católicos, 2, 28040 Madrid, Spain; 2 Mosaiques Diagnostics GmbH, Rotenburger Str. 20, 30659 Hannover, Germany; 3 Nephrology Service, Reina Sofía University Hospital/University of Córdoba. Instituto Maimónides de Investigación Biomédica de Córdoba (IMIBIC). Avda. Menéndez Pidal, S/N. 14004, Córdoba(Spain). REDinREN, Madrid, Spain; 4 Institute of Cardiovascular and Medical Sciences, 126 University Pl, G12 8TA Glasgow, United Kingdom

Correction to: *Scientific Reports* 10.1038/s41598-018-34386-8, published online 29 October 2018

The original version of this Article contained an error in the title of the paper, where the word “eGFR” was incorrectly given as “Egfr”

This has now been corrected in the PDF and HTML versions of the Article.

