# Supplementary material for: Novel Urinary Biomarkers For Improved Prediction Of Progressive eGFR Loss In Early Chronic Kidney Disease Stages And In High Risk Individuals Without Chronic Kidney Disease
Source: Sci Rep. 2018 Oct 29;8:15940. doi: 10.1038/s41598-018-34386-8 (PMC6206033; doi:10.1038/s41598-018-34386-8)
Supplement: Supplementary file 1 — Supplementary information [file 41598_2018_34386_MOESM1_ESM.doc]

**Novel urinary biomarkers for improved prediction of progressive eGFR loss in early chronic kidney disease stages and in high risk individuals without chronic kidney disease**

María E. Rodríguez-Ortiz PhD, Claudia Pontillo PhD, Mariano Rodríguez MD PhD, Petra Zürbig PhD, Harald Mischak PhD, Alberto Ortiz MD PhD

**SUPPLEMENTARY MATERIAL**

**KFRE equation**

KFRE equation is as follows:

*p* = 1 - *S_ave_* (*t* = 1826)*^a^*

*a* = -0.55418 x [(*eGFR*/5) – 7.22] + 0.26940 x (*male* – 0.56) + 0.45608 x [*ln* (*ACR*) – 5.2774] – 0.21670 x [(*age*/10) – 7.04]

where:

p: risk of kidney failure

S_ave_ (t = 1826): survival rate for an individual with the average value of covariates in the risk equation

eGFR: estimated glomerular filtration rate, expressed as ml/min/1.73 m^2^.

ln (ACR): natural logarithm of albumin-creatinine ratio (expressed as mg/g).

male: indicator for sex (male = 1, female = 0).

age: age (years) at test date.

**Correlations of the continuous variable “eGFR decline” with the CKD273 classifier and the CKD273 subclassifiers**

In addition to considering “rapid progression” as a dichotomous variable, we examined the correlation between the continuous variable “eGFR decline” and the CKD273 classifier score and, on the other hand, between “eGFR decline” and the CKD273 subclassifier scores. The rate of eGFR decline and CKD273 classifier values exhibited an inverse correlation that was significant in patients with eGFR ≥30 ml/min/1.73 m^2^. After adjusting for albuminuria or baseline eGFR, the association remained significant for patients with eGFR ≥70 ml/min/1.73 m^2^ or ≥30 ml/min/1.73 m^2^, respectively (table S12). The association between eGFR decline and CKD273 subclassifier scores with best AUC in the validation set was also inverse and significant for all ranges of eGFR. This relationship remained significant after adjusting by albuminuria in subjects with eGFR ≥40 ml/min/1.73 m^2^, whereas baseline eGFR had no influence at any eGFR stratum (table S13).

**Analysis of the peptides most differentially expressed in each CKD273 subclassifier**

In patients with early CKD (eGFR ≥60 ml/min/1.73 m^2^), differential peptides between rapid progressors and non-rapid progressors were predominantly collagen fragments, which were down-regulated in rapid progressors and represented 86% of the total number of biomarkers in strata 1 to 3 (figure 3A). In particular, collagen alpha-1(I) peptides were the most abundant, constituting 67% of the most differentially expressed peptides. In addition, uromodulin (stratum 1) and sodium/potassium-transporting ATPase subunit gamma (stratum 2) peptides were also significantly down-regulated in rapid progressors. In more advanced CKD (eGFR ≤50 ml/min/1.73 m^2^), peptides belonged to a distinct pattern of proteins (figure 3A). Collagen fragments still represented 70% of the most differentially expressed peptides in strata 5 and 7. In addition, in strata 4 to 7, the expression of peptides derived from serum proteins, like alpha-1-antitrypsin, C3 complement, albumin, fibrinogen alpha or transport proteins (retinol-binding protein 4, or several apolipoproteins) was higher in rapid progressors. Table S11 shows the fold change of differentially expressed peptides in patients with faster CKD progression.

Further, we assessed which peptides were also present in the CKD273 classifier (table 4 and figure 3B). The highest number of common peptides between the CKD273 subclassifiers and the CKD273 classifier was observed in stratum 1 (7 out of the 10 top differentially expressed peptides), whereas in stratum 6 there were no peptides overlapping with the CKD273 classifier. The majority of the overlapping peptides were collagen fragments, particularly collagen alpha-1(I) (strata 1, 2, 3, and 5). Additional peptides overlapping with the CDK273 classifier belonged to the sodium/potassium-transporting ATPase subunit gamma and uromodulin (stratum 1), two alpha-1-antitrypsin peptides (stratum 4), and fibrinogen alpha chain and serum albumin (stratum 5).

**Table S1. Overview of the training and validation sets for each of the generated CKD273 subclassifiers. The table is read as follows: for the CKD273 subclassifier developed in eGFR stratum 1, the training/discovery set were the patients in eGFR stratum 1 (eGFR ≥80 ml/min/1.73 m^2^) and six different eGFR strata (stratum 2 through stratum 7) were used individually as validation sets.**

|  | **STRATUM WHERE CKD273 SUBCLASSIFIER WAS DEVELOPED** | | | | | | |
| --- | --- | --- | --- | --- | --- | --- | --- |
|  | **STRATUM 1** | **STRATUM 2** | **STRATUM 3** | **STRATUM 4** | **STRATUM 5** | **STRATUM 6** | **STRATUM 7** |
| **STRATUM 1** | **Training set** | Validation set | Validation set | Validation set | Validation set | Validation set | Validation set |
| **STRATUM 2** | Validation set | **Training set** | Validation set | Validation set | Validation set | Validation set | Validation set |
| **STRATUM 3** | Validation set | Validation set | **Training set** | Validation set | Validation set | Validation set | Validation set |
| **STRATUM 4** | Validation set | Validation set | Validation set | **Training set** | Validation set | Validation set | Validation set |
| **STRATUM 5** | Validation set | Validation set | Validation set | Validation set | **Training set** | Validation set | Validation set |
| **STRATUM 6** | Validation set | Validation set | Validation set | Validation set | Validation set | **Training set** | Validation set |
| **STRATUM 7** | Validation set | Validation set | Validation set | Validation set | Validation set | Validation set | **Training set** |

**Table S2. Prediction of rapid CKD progression. Area under the curve (AUC) and 95% confidence interval (CI) of the performance in the validation cohorts of the CKD273 subclassifiers generated for each stratum. eGFR expressed as ml/min/1.73 m^2^.**

| **eGFR stratum where CKD273 subclassifier was generated (training set)** | **eGFR strata where CKD273 subclassifier was validated** | **AUC (95% CI) in validation strata**  **(*P<0.05)** |
| --- | --- | --- |
| ≥80 | 70-79 | 0.736 (0.689-0.780)* |
|  | 60-69 | 0.741 (0.683-0.793)* |
|  | 50-59 | 0.693 (0.605-0.772)* |
|  | 40-49 | 0.644 (0.517-0.757)* |
|  | 30-39 | 0.527 (0.412-0.639) |
|  | <30 | 0.571 (0.461-0.676) |
| 70-79 | >80 | 0.782 (0.743-0.819)* |
|  | 60-69 | 0.797 (0.743-0.844)* |
|  | 50-59 | 0.692 (0.603-0.771)* |
|  | 40-49 | 0.711 (0.587-0.815)* |
|  | 30-39 | 0.532 (0.417-0.644) |
|  | <30 | 0.648 (0.539-0.747)* |
| 60-69 | >80 | 0.778 (0.738-0.815)* |
|  | 70-79 | 0.751 (0.705-0.794)* |
|  | 50-59 | 0.723 (0.637-0.799)* |
|  | 40-49 | 0.764 (0.644-0.859)* |
|  | 30-39 | 0.637 (0.522-0.742)* |
|  | <30 | 0.730 (0.625-0.819)* |
| 50-59 | >80 | 0.555 (0.510-0.601) |
|  | 70-79 | 0.575 (0.524-0.626) |
|  | 60-69 | 0.560 (0.498-0.621) |
|  | 40-49 | 0.824 (0.712-0.906)* |
|  | 30-39 | 0.629 (0.513-0.734)* |
|  | <30 | 0.547 (0.437-0.653) |
| **eGFR stratum where CKD273 subclassifier was generated (training set)** | **eGFR strata where CKD273 subclassifier was validated** | **AUC (95% CI) in validation strata** |
| 40-49 | >80 | 0.538 (0.493-0.584) |
|  | 70-79 | 0.546 (0.495-0.597) |
|  | 60-69 | 0.642 (0.580-0.700)* |
|  | 50-59 | 0.728 (0.641-0.803)* |
|  | 30-39 | 0.707 (0.595-0.804)* |
|  | <30 | 0.688 (0.581-0.783)* |
| 30-39 | >80 | 0.624 (0.579-0.668)* |
|  | 70-79 | 0.615 (0.564-0.664)* |
|  | 60-69 | 0.539 (0.476-0.601) |
|  | 50-59 | 0.655 (0.565-0.737)* |
|  | 40-49 | 0.728 (0.605-0.830)* |
|  | <30 | 0.646 (0.537-0.745)* |
| <30 | >80 | 0.514 (0.469-0.560) |
|  | 70-79 | 0.500 (0.449-0.552) |
|  | 60-69 | 0.605 (0.543-0.664) |
|  | 50-59 | 0.602 (0.511-0.688) |
|  | 40-49 | 0.690 (0.566-0.798)* |
|  | 30-39 | 0.690 (0.577-0.789)* |

**Table S3. CKD273 subclassifiers scores for the best performing CKD273 subclassifier in the validation set in rapid progressors and non-rapid progressors in different baseline eGFR strata. For patients with baseline eGFR (≥80 ml/min/1.73 m^2^), the best predictor of rapid progression in the validation set was the CKD273 subclassifier generated in the 60-69 ml/min/1.73 m^2^ training stratum.**

| **Stratum (baseline eGFR)** | **Best performing CKD273 subclassifier in the validation set** | **CKD273 subclassifier score. Rapid progressors** | **CKD273 subclassifier score. Non-rapid progressors** | **P-value** |
| --- | --- | --- | --- | --- |
| **1 (≥80 ml/min/1.73 m^2^)** | 60-69 CKD273 subclassifier | -0.1218±0.641 | -0.7422±0.515 | <0.001 |
| **2 (70-79 ml/min/1.73 m^2^)** | 60-69 CKD273 subclassifier | -0.0579±0.567 | -0.5767±0.545 | <0.001 |
| **3 (60-69 ml/min/1.73 m^2^)** | ≥80 CKD273 subclassifier | 0.8150±0.963 | 0.0074±0.902 | <0.001 |
| **4 (50-59 ml/min/1.73 m^2^)** | 40-49 CKD273 subclassifier | -0.0713±1.028 | -0.9188±0.436 | <0.01 |
| **5 (40-49 ml/min/1.73 m^2^)** | 50-59 CKD273 subclassifier | 0.3637±0.669 | -0.5867±0.828 | <0.001 |
| **6 (30-39 ml/min/1.73 m^2^)** | 40-49 CKD273 subclassifier | 0.6723±0.517 | 0.2528±0.576 | <0.01 |
| **7 (<30 ml/min/1.73 m^2^)** | 30-39 CKD273 subclassifier | 0.5902±0.972 | 0.0333±0.883 | <0.05 |

Data expressed as mean ± standard deviation.

**Table S4. Scores for the risk of renal failure according to the KFRE equation at 2 and 5 years in rapid progressors and non-rapid progressors in baseline eGFR strata.**

| **Stratum (baseline eGFR)** | **Time** | **KFRE risk rapid progressors (%)** | **KFRE risk non-rapid progressors (%)** | **P-value** |
| --- | --- | --- | --- | --- |
| **1 (≥80 ml/min/1.73 m^2^)** | 2-year | 0.0007±0.003 | 0.0001±0.001 | <0.05 |
|  | 5-year | 0.0067±0.017 | 0.0075±0.008 | <0.001 |
| **2 (70-79 ml/min/1.73 m^2^)** | 2-year | 0.0063±0.013 | 0.0057±0.007 | n.s. |
|  | 5-year | 0.0383±0.046 | 0.0379±0.248 | <0.01 |
| **3 (60-69 ml/min/1.73 m^2^)** | 2-year | 0.0336±0.053 | 0.0219±0.031 | n.s. |
|  | 5-year | 0.1408±0.210 | 0.0989±0.122 | n.s. |
| **4 (50-59 ml/min/1.73 m^2^)** | 2-year | 0.6019±0668 | 0.0825±0.191 | <0.01 |
|  | 5-year | 2.2988±2.524 | 0.3308±0.724 | <0.05 |
| **5 (40-49 ml/min/1.73 m^2^)** | 2-year | 2.2504±1.246 | 0.8205±1.045 | <0.001 |
|  | 5-year | 8.3704±4.440 | 3.0988±3.886 | <0.001 |
| **6 (30-39 ml/min/1.73 m^2^)** | 2-year | 7.3586±3.721 | 4.9192±2.974 | <0.01 |
|  | 5-year | 24.9925±11.070 | 17.3256±9.815 | <0.01 |
| **7 (<30 ml/min/1.73 m^2^)** | 2-year | 19.6229±7.658 | 17.5808±9.251 | n.s. |
|  | 5-year | 55.0361±15.212 | 49.6016±19.140 | n.s. |

Data expressed as mean ± standard deviation.

**Table S5. Prediction of rapid CKD progression in individuals without CKD (i.e. those with eGFR ≥60 ml/min/1.73 m^2^ and UAE <30 mg/24h). Area under the curve (AUC) and 95% confidence interval (95% CI) for CKD273 subclassifiers (identified as the eGFR strata where they were developed) in validation sets, compared with albuminuria and CKD273 classifier. eGFR expressed as ml/min/1.73 m^2^.**

| **Validation eGFR stratum (eGFR)** | **CKD273 subclassifier*** | **AUC (95% CI) in validation strata** | **P-value (vs albuminuria)** | **P-value (vs CKD273)** |
| --- | --- | --- | --- | --- |
| 1 (≥80) | 70-79 | 0.782 (0.743-0.819) | <0.001 | <0.01 |
|  | 60-69 | 0.778 (0.738-0.815) | <0.001 | <0.01 |
|  | 50-59 | 0.555 (0.510-0.601) | <0.05 | <0.001 |
|  | 40-49 | 0.538 (0.493-0.584) | <0.05 | <0.001 |
|  | 30-39 | 0.624 (0.579-0.668) | 0.78 | <0.05 |
|  | <30 | 0.514 (0.469-0.560) | <0.01 | <0.001 |
| 2 (70-79) | ≥80 | 0.736 (0.689-0.780) | <0.001 | 0.48 |
|  | 60-69 | 0.751 (0.705-0.794) | <0.001 | 0.16 |
|  | 50-59 | 0.575 (0.524-0.626) | 0.90 | <0.05 |
|  | 40-49 | 0.546 (0.495-0.597) | 0.68 | <0.001 |
|  | 30-39 | 0.615 (0.564-0.664) | 0.40 | 0.06 |
|  | <30 | 0.500 (0.449-0.552) | 0.26 | <0.001 |
| 3 (60-69) | ≥80 | 0.741 (0.683-0.793) | 0.51 | 0.41 |
|  | 70-79 | 0.797 (0.743-0.844) | 0.07 | <0.05 |
|  | 50-59 | 0.560 (0.498-0.621) | 0.07 | 0.11 |
|  | 40-49 | 0.642 (0.580-0.700) | 0.60 | 0.60 |
|  | 30-39 | 0.539 (0.476-0.601) | 0.10 | 0.10 |
|  | <30 | 0.605 (0.543-0.664) | 0.32 | 0.24 |

*****Column represents the eGFR stratum where the CKD273 subclassifier was generated in the training set.

**Table S6. Prediction of rapid CKD progression in individuals with eGFR ≥60 ml/min/1.73 m^2^ (validation set).**

**Model 1: subclassifier adjusted for albuminuria, DM, and eGFR.**

**Model 2: subclassifier adjusted for albuminuria, DM, eGFR, sex, age, and systolic blood pressure.**

|  | **Model 1** | | **Model 2** | |
| --- | --- | --- | --- | --- |
|  | **HR (95% CI)** | **p-value** | **HR (95% CI)** | **P-value** |
| **eGFR stratum 1 (eGFR >80 ml/min/1.73 m^2^):** | | | | |
| Stratum 2 CKD273 subclassifier | 1.91 (1.56 to 2.35) | <0.0001 | 1.33 (1.01 to 1.75) | 0.0427 |
| CKD273 | 1.96 (1.27 to 3.02) | 0.0024 | 1.10 (0.75 to 1.67) | 0.6783 |
| **eGFR stratum 2 (eGFR 70-79 ml/min/1.73 m^2^):** | | | | |
| Stratum 1 CKD273 subclassifier | 2.19 (1.62 to 2.96) | <0.0001 | 2.14 (1.52 to 3.02) | <0.0001 |
| CKD273 | 4.08 (2.17 to 7.69) | <0.0001 | 3.46 (1.75 to 6.86) | 0.0004 |
| **eGFR stratum 3 (eGFR 60-69 ml/min/1.73 m^2^):** | | | | |
| Stratum 2 CKD273 subclassifier | 4.57 (2.17 to 9.59) | 0.0001 | 4.25 (1.67 to 10.81) | 0.0025 |
| CKD273 | 2.26 (0.83 to 6.12) | 0.1120 | 2.10 (0.62 to 7.16) | 0.2383 |

**Table S7. Prediction of rapid CKD progression in individuals with eGFR ≥60 ml/min/1.73 m^2^ and UAE <30 mg/24h (validation set).**

**Model 1: subclassifier adjusted for albuminuria, DM, and eGFR.**

**Model 2: subclassifier adjusted for albuminuria, DM, eGFR, sex, age, and systolic blood pressure.**

|  | **Model 1** | | **Model 2** | |
| --- | --- | --- | --- | --- |
|  | **HR (95% CI)** | **p-value** | **HR (95% CI)** | **P-value** |
| **eGFR stratum 1 (eGFR >80 ml/min/1.73 m^2^):** | | | | |
| Stratum 2 CKD273 subclassifier | 1.83 (1.49 to 2.24) | <0.0001 | 1.30 (0.98 to 1.71) | 0.0687 |
| CKD273 | 1.64 (1.05 to 2.56) | 0.0308 | 1.06 (0.65 to 1.47) | 0.2919 |
| **eGFR stratum 2 (eGFR 70-79 ml/min/1.73 m^2^):** | | | | |
| Stratum 1 CKD273 subclassifier | 1.97 (1.44 to 2.68) | <0.0001 | 1.72 (1.20 to 2.48) | 0.0037 |
| CKD273 | 3.14 (1.61 to 6.12) | 0.0008 | 2.25 (1.11 to 4.57) | 0.0256 |
| **eGFR stratum 3 (eGFR 60-69 ml/min/1.73 m^2^):** | | | | |
| Stratum 2 CKD273 subclassifier | 4.08 (1.88 to 8.83) | 0.0004 | 2.91 (1.14 to 7.43) | 0.0264 |
| CKD273 | 1.64 (0.47 to 5.71) | 0.4372 | 1.56 (0.39 to 6.30) | 0.2383 |

**Table S8. Bivariate correlation coefficients of the best validation model scores and eGFR decline per each stratum in non-rapid progressors. Coefficients after adjusting by diabetes are also shown.**

| **eGFR stratum** | **Correlation coefficient** | **Adjusted by diabetes** |
| --- | --- | --- |
| **1** | -0.025* | -0.066* |
| **2** | -0.084* | -0.134 |
| **3** | -0.055* | -0.163 |
| **4** | -0.062* | -0.021* |
| **5** | 0.008* | -0.066* |
| **6** | 0.001* | -0.108* |
| **7** | 0.078* | 0.084* |

*P-value <0.05

**Table S9. Prediction of rapid CKD progression in diabetic individuals with eGFR ≥60 ml/min/1.73 m^2^ (validation set).**

**Model 1: subclassifier adjusted for albuminuria, and eGFR.**

**Model 2: subclassifier adjusted for albuminuria, eGFR, sex, age, and systolic blood pressure.**

|  | **Model 1** | | **Model 2** | |
| --- | --- | --- | --- | --- |
|  | **HR (95% CI)** | **p-value** | **HR (95% CI)** | **P-value** |
| **eGFR stratum 1 (eGFR >80 ml/min/1.73 m^2^):** | | | | |
| Stratum 2 CKD273 subclassifier | 1.86 (1.52 to 2.28) | <0.0001 | 1.27 (0.96 to 1.69) | 0.097 |
| CKD273 | 1.79 (1.14 to 2.80) | 0.011 | 0.73 (0.43 to 1.24) | 0.250 |
| **eGFR stratum 2 (eGFR 70-79 ml/min/1.73 m^2^):** | | | | |
| Stratum 1 CKD273 subclassifier | 2.20 (1.60 to 3.02) | <0.0001 | 1.84 (1.30 to 2.62) | 0.001 |
| CKD273 | 3.64 (1.84 to 7.20) | <0.0001 | 2.33 (1.16 to 4.66) | 0.017 |
| **eGFR stratum 3 (eGFR 60-69 ml/min/1.73 m^2^):** | | | | |
| Stratum 2 CKD273 subclassifier | 5.09 (2.30 to 11.31) | <0.0001 | 4.22 (1.58 to 11.25) | 0.004 |
| CKD273 | 2.22 (0.75 to 6.58) | 0.152 | 2.30 (0.61 to 8.60) | 0.217 |

**Table S10. Prediction of rapid CKD progression in diabetic individuals with eGFR ≥60 ml/min/1.73 m^2^ and UAE <30 mg/24h (validation set).**

**Model 1: subclassifier adjusted for albuminuria, and eGFR.**

**Model 2: subclassifier adjusted for albuminuria, eGFR, sex, age, and systolic blood pressure.**

|  | **Model 1** | | **Model 2** | |
| --- | --- | --- | --- | --- |
|  | **HR (95% CI)** | **p-value** | **HR (95% CI)** | **P-value** |
| **eGFR stratum 1 (eGFR >80 ml/min/1.73 m^2^):** | | | | |
| Stratum 2 CKD273 subclassifier | 1.86 (1.52 to 2.29) | <0.0001 | 1.32 (0.99 to 1.75) | 0.060 |
| CKD273 | 1.74 (1.10 to 2.74) | 0.018 | 0.76 (0.44 to 1.30) | 0.315 |
| **eGFR stratum 2 (eGFR 70-79 ml/min/1.73 m^2^):** | | | | |
| Stratum 1 CKD273 subclassifier | 2.08 (1.50 to 2.88) | <0.0001 | 1.64 (1.14 to 2.36) | 0.008 |
| CKD273 | 3.96 (1.95 to 8.02) | <0.0001 | 2.28 (1.11 to 4.69) | 0.026 |
| **eGFR stratum 3 (eGFR 60-69 ml/min/1.73 m^2^):** | | | | |
| Stratum 2 CKD273 subclassifier | 4.64 (2.03 to 10.59) | <0.0001 | 2.95 (1.16 to 7.49) | 0.023 |
| CKD273 | 1.55 (0.40 to 6.05) | 0.529 | 1.55 (0.38 to 6.36) | 0.546 |

**Table S11. Fold change and regulation of the ten peptides most differentially expressed between rapid progressors and non-rapid progressors in each eGFR stratum. Several different peptides may map for a unique protein (protein name provided in the table). Peptides are ordered according to the magnitude of their differential expression between rapid progressors and non-rapid progressors.**

| **eGFR stratum** | **Protein name** | **Fold change*** | **Regulation (in rapid progressors)** |
| --- | --- | --- | --- |
| 1 | Uromodulin | 0.35 | Down-regulated |
|  | Uromodulin | 0.31 | Down-regulated |
|  | Collagen alpha-1(I) chain | 0.47 | Down-regulated |
|  | Collagen alpha-1(I) chain | 0.51 | Down-regulated |
|  | Collagen alpha-1(I) chain | 0.55 | Down-regulated |
|  | Collagen alpha-1(I) chain | 0.56 | Down-regulated |
|  | Collagen alpha-1(I) chain | 0.58 | Down-regulated |
|  | Sodium/potassium-transporting ATPase subunit gamma | 0.42 | Down-regulated |
|  | Collagen alpha-1(I) chain | 0.60 | Down-regulated |
|  | Collagen alpha-1(I) chain | 0.63 | Down-regulated |
| 2 | Collagen alpha-1(I) chain | 0.47 | Down-regulated |
|  | Collagen alpha-1(I) chain | 0.45 | Down-regulated |
|  | Collagen alpha-2(I) chain | 0.38 | Down-regulated |
|  | Collagen alpha-1(I) chain | 0.37 | Down-regulated |
|  | Collagen alpha-1(I) chain | 0.54 | Down-regulated |
|  | Collagen alpha-1(I) chain | 0.50 | Down-regulated |
|  | Collagen alpha-2(I) chain | 0.51 | Down-regulated |
|  | Collagen alpha-1(I) chain | 0.56 | Down-regulated |
|  | Sodium/potassium-transporting ATPase subunit gamma | 0.46 | Down-regulated |
|  | Collagen alpha-1(II) chain | 0.59 | Down-regulated |
| 3 | Collagen alpha-1(I) chain | 0.12 | Down-regulated |
|  | Collagen alpha-1(I) chain | 0.34 | Down-regulated |
|  | Collagen alpha-1(I) chain | 0.34 | Down-regulated |
|  | Collagen alpha-1(I) chain | 0.57 | Down-regulated |
|  | Collagen alpha-1(I) chain | 0.22 | Down-regulated |
|  | Collagen alpha-1(III) chain | 0.41 | Down-regulated |
|  | Collagen alpha-1(I) chain | 0.14 | Down-regulated |
|  | Collagen alpha-1(I) chain | 0.44 | Down-regulated |
|  | Collagen alpha-1(XIV) chain | 0.45 | Down-regulated |
|  | Collagen alpha-2(I) chain | 0.21 | Down-regulated |
| 4 | Alpha-1-antitrypsin | 41.48 | Up-regulated |
|  | Alpha-1-antitrypsin | 28.29 | Up-regulated |
|  | Retinol-binding protein 4 | 79.01 | Up-regulated |
|  | Alpha-1-antitrypsin | 63.80 | Up-regulated |
|  | Apolipoprotein A-IV | 532.56 | Up-regulated |
|  | Alpha-1-antitrypsin | 295.69 | Up-regulated |
|  | Serum paraoxonase/arylesterase 1 | 342.86 | Up-regulated |
|  | Alpha-1B-glycoprotein | 10.69 | Up-regulated |
|  | Cornulin | 2.17 | Up-regulated |
|  | Complement C3 | 27.59 | Up-regulated |
| 5 | Collagen alpha-1(I) chain | 0.31 | Down-regulated |
|  | Collagen alpha-1(I) chain | 0.16 | Down-regulated |
|  | Collagen alpha-1(XIV) chain | 0.12 | Down-regulated |
|  | Collagen alpha-1(I) chain | 0.24 | Down-regulated |
|  | Fibrinogen alpha chain | 3.68 | Up-regulated |
|  | Collagen alpha-1(I) chain | 0.48 | Down-regulated |
|  | Collagen alpha-1(I) chain | 0.36 | Down-regulated |
|  | Collagen alpha-2(IX) chain | 0.28 | Down-regulated |
|  | Serum albumin | 14.87 | Up-regulated |
|  | Collagen alpha-1(II) chain | 0.23 | Down-regulated |
| 6 | Apolipoprotein A-IV | 1.55 | Up-regulated |
|  | Collagen alpha-1(II) chain | 0.51 | Down-regulated |
|  | Collagen alpha-1(XIX) chain | 1.44 | Up-regulated |
|  | Antithrombin-III | 2.87 | Up-regulated |
|  | Unconventional myosin-Ib | 5.23 | Up-regulated |
|  | Apolipoprotein A-I | 9.69 | Up-regulated |
|  | Beta-2-microglobulin form pI 5.3 | 5.27 | Up-regulated |
|  | Beta-2-microglobulin form pI 5.3 | 6.62 | Up-regulated |
|  | Peptidase inhibitor 16 | 3.58 | Up-regulated |
|  | Complement C3 | 2.01 | Up-regulated |
| 7 | Collagen alpha-1(I) chain | 0.44 | Down-regulated |
|  | Collagen alpha-1(III) chain | 0.41 | Down-regulated |
|  | Collagen alpha-1(III) chain | 0.47 | Down-regulated |
|  | Serum amyloid A-2 protein | 0.46 | Down-regulated |
|  | T-lymphoma invasion and metastasis-inducing protein 1 | 0.38 | Down-regulated |
|  | Collagen alpha-1(I) chain | 0.40 | Down-regulated |
|  | Collagen alpha-1(XVI) chain | 0.43 | Down-regulated |
|  | Collagen alpha-2(V) chain | 0.48 | Down-regulated |
|  | Calgranulin B | 0.47 | Down-regulated |
|  | Collagen alpha-1(I) chain | 0.36 | Down-regulated |

**Table S12. Correlation coefficients of the continuous variable “eGFR decline” and the CKD273 classifier score per each eGFR stratum, and after adjusting by albuminuria or baseline eGFR expressed as ml/min/1.73 m^2^**.

| **eGFR stratum** | **Correlation coefficient** | **Adjusted by albuminuria** | **Adjusted by baseline eGFR** |
| --- | --- | --- | --- |
| **>80** | -0.351* | -0.273* | -0.392* |
| **70-79** | -0.297* | -0.297* | -0.301* |
| **60-69** | -0.216* | -0.095 | -0.216* |
| **50-59** | -0.424* | -0.068 | -0.413* |
| **40-49** | -0.322* | 0.211 | -0.365* |
| **30-39** | -0.168 | -0.040 | -0.180 |
| **<30** | 0.061 | 0.083 | 0.044 |

*P-value <0.05

**Table S13. Correlation coefficients of the continuous variable “eGFR decline” and the scores for the CKD273 subclassifiers with higher AUC in the validation set for each eGFR stratum, and after adjusting by albuminuria or baseline eGFR expressed as ml/min/1.73 m^2^**.

| **eGFR stratum** | **Correlation coefficient** | **Adjusted by albuminuria** | **Adjusted by baseline eGFR** |
| --- | --- | --- | --- |
| **>80** | -0.421* | -0.367* | -0.448* |
| **70-79** | -0.300* | -0.282* | -0.302* |
| **60-69** | -0.213* | -0.157* | -0.216* |
| **50-59** | -0.587* | -0.228* | -0.583* |
| **40-49** | -0.471* | -0.067 | -0.492* |
| **30-39** | -0.367* | -0.163 | -0.418* |
| **<30** | -0.271* | -0.146 | -0.273* |

*P-value <0.05

**SUPPLEMENTARY FIGURE LEGEND**

**Supplementary figure 1. Distribution of CKD273 subclassifier scores for rapid and non-rapid progressors in each baseline eGFR stratum.** Data for the CKD273 subclassifier best performing in the validation set are shown. Median and quartiles are shown below the figures.

**Supplementary figure 2. ROC curves for the CKD273 subclassifier predicting rapid CKD progression in each baseline eGFR stratum.** Data for the CKD273 subclassifier best performing in the validation set are shown. Sensitivity, specificity and positive and negative predictive values are shown below the graphs.

**Supplementary figure 3. Distribution of the Kidney Failure Risk Equation (KFRE) estimated risk of progression to end-stage renal disease at 2 years in rapid and non-rapid progressors in each baseline eGFR stratum.** Median and quartiles are shown below the figures.

**Supplementary figure 4. Distribution of the Kidney Failure Risk Equation (KFRE) estimated risk of progression to end-stage renal disease at 5 years in rapid and non-rapid progressors in each baseline eGFR stratum.** Median and quartiles are shown below the figures.

**Supplementary figure 5. ROC curves for the Kidney Failure Risk Equation (KFRE) estimated risk of progression to end-stage renal disease at 2 years in each baseline eGFR stratum.** Sensitivity, specificity and positive and negative predictive values are shown below the graphs.

**Supplementary figure 6. ROC curves for the Kidney Failure Risk Equation (KFRE) estimated risk of progression to end-stage renal disease at 5 years in each baseline eGFR stratum.** Sensitivity, specificity and positive and negative predictive values are shown below the graphs.

**SUPPLEMENTARY FIGURE 1**

**
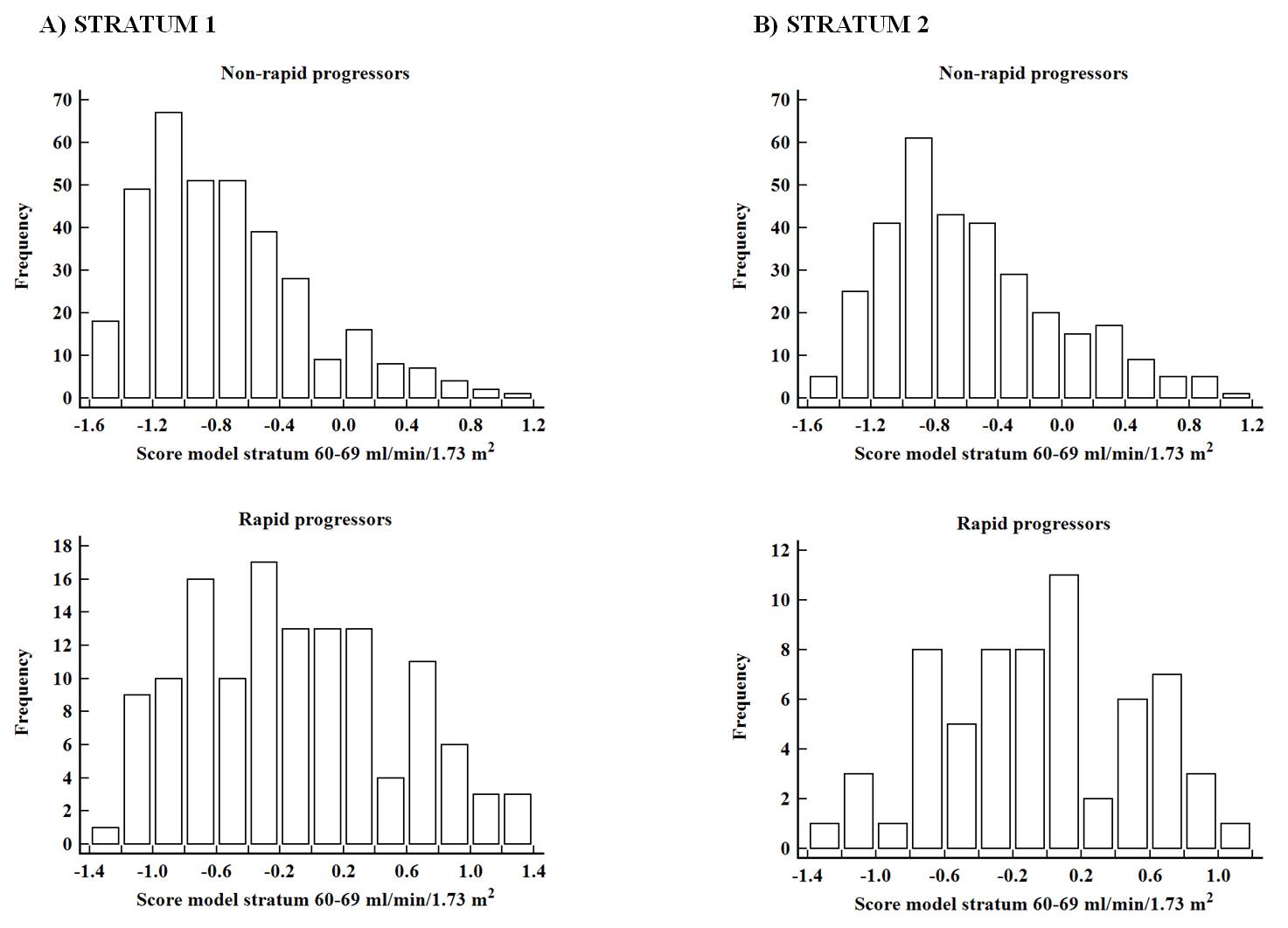
**

**
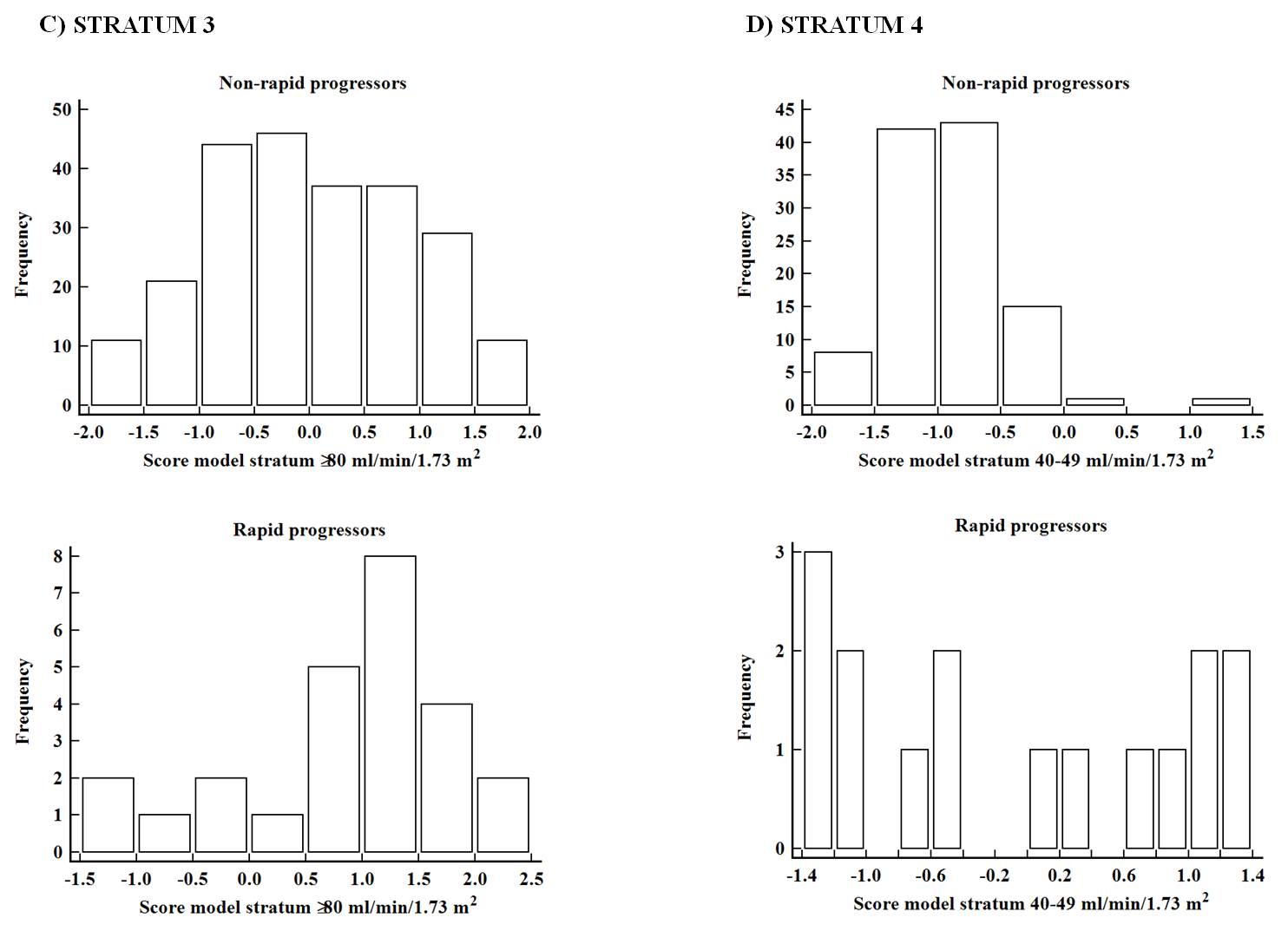
**

**
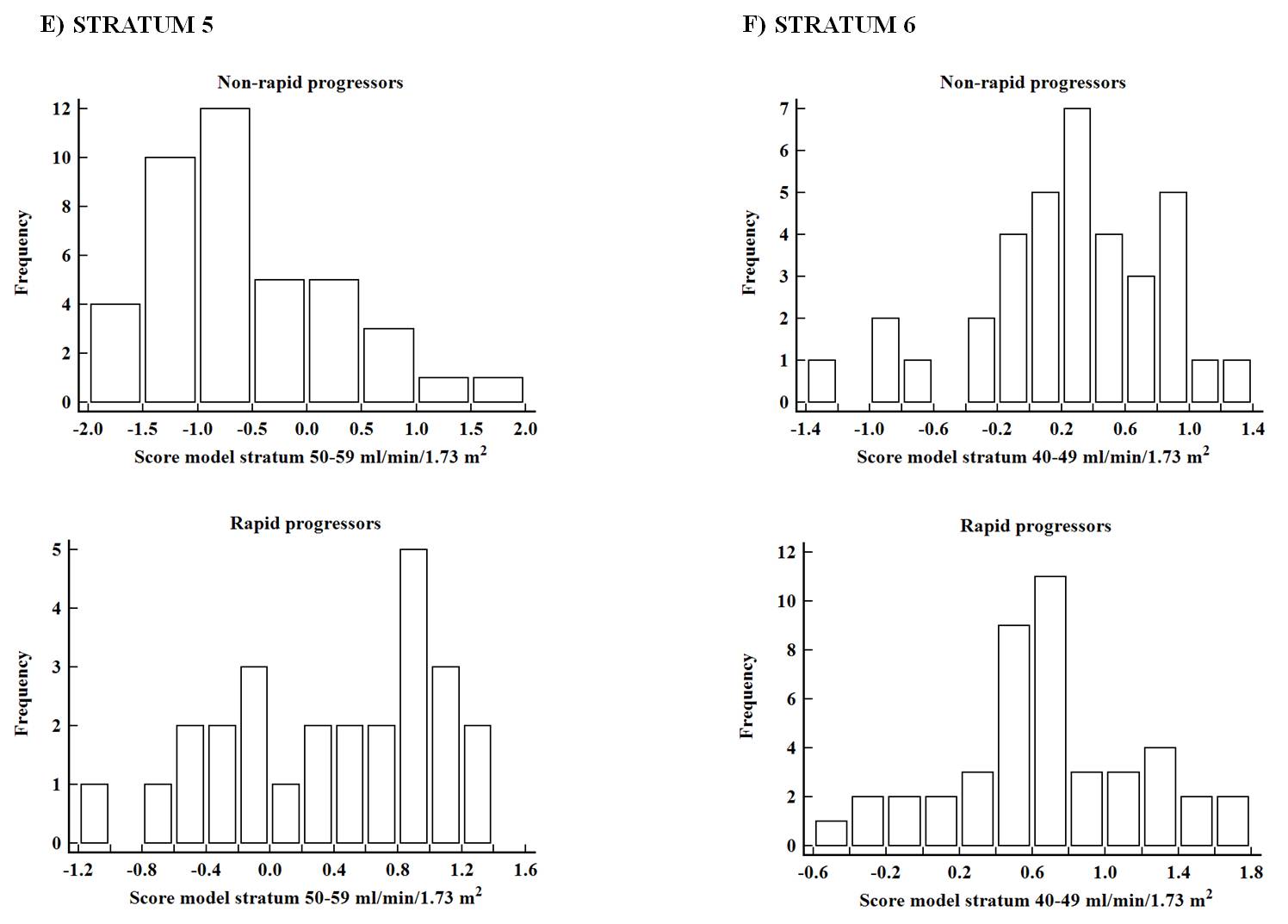
**

**
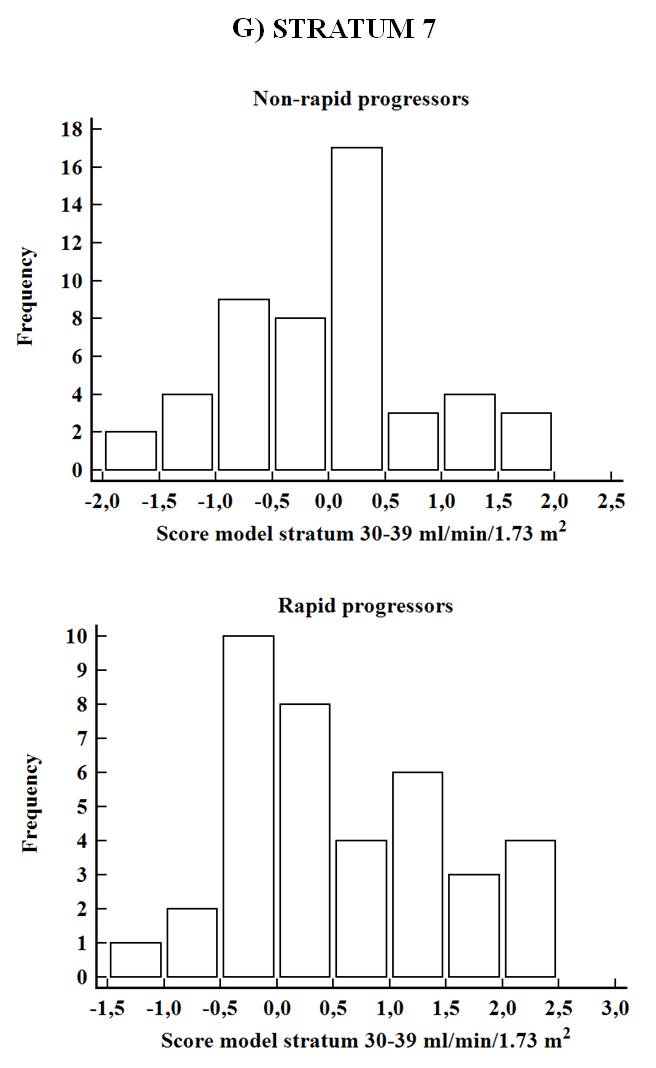
**

|  | **Rapid progressors** | | | **Non-rapid progressors** | | |
| --- | --- | --- | --- | --- | --- | --- |
| **Stratum (baseline eGFR)** | **Q25** | **Q50** | **Q75** | **Q25** | **Q50** | **Q75** |
| **1 (≥80 ml/min/1.73 m^2^)** | -0.641 | -0.187 | 0.312 | -1.130 | -0.827 | -0.485 |
| **2 (70-79 ml/min/1.73 m^2^)** | -0.460 | -0.029 | 0.445 | -0.966 | -0.685 | -0.250 |
| **3 (60-69 ml/min/1.73 m^2^)** | 0.526 | 1.038 | 1.435 | -0.737 | -0.020 | 0.671 |
| **4 (50-59 ml/min/1.73 m^2^)** | -1.044 | -0.200 | 1.056 | -1.219 | -0.951 | -0.673 |
| **5 (40-49 ml/min/1.73 m^2^)** | -0.213 | 0.495 | 0.914 | -1.251 | -0.767 | -0.074 |
| **6 (30-39 ml/min/1.73 m^2^)** | 0.426 | 0.643 | 1.068 | -0.086 | 0.320 | 0.627 |
| **7 (<30 ml/min/1.73 m^2^)** | -0.212 | 0.372 | 1.366 | -0.596 | 0.045 | 0.465 |

**SUPPLEMENTARY FIGURE 2**

**
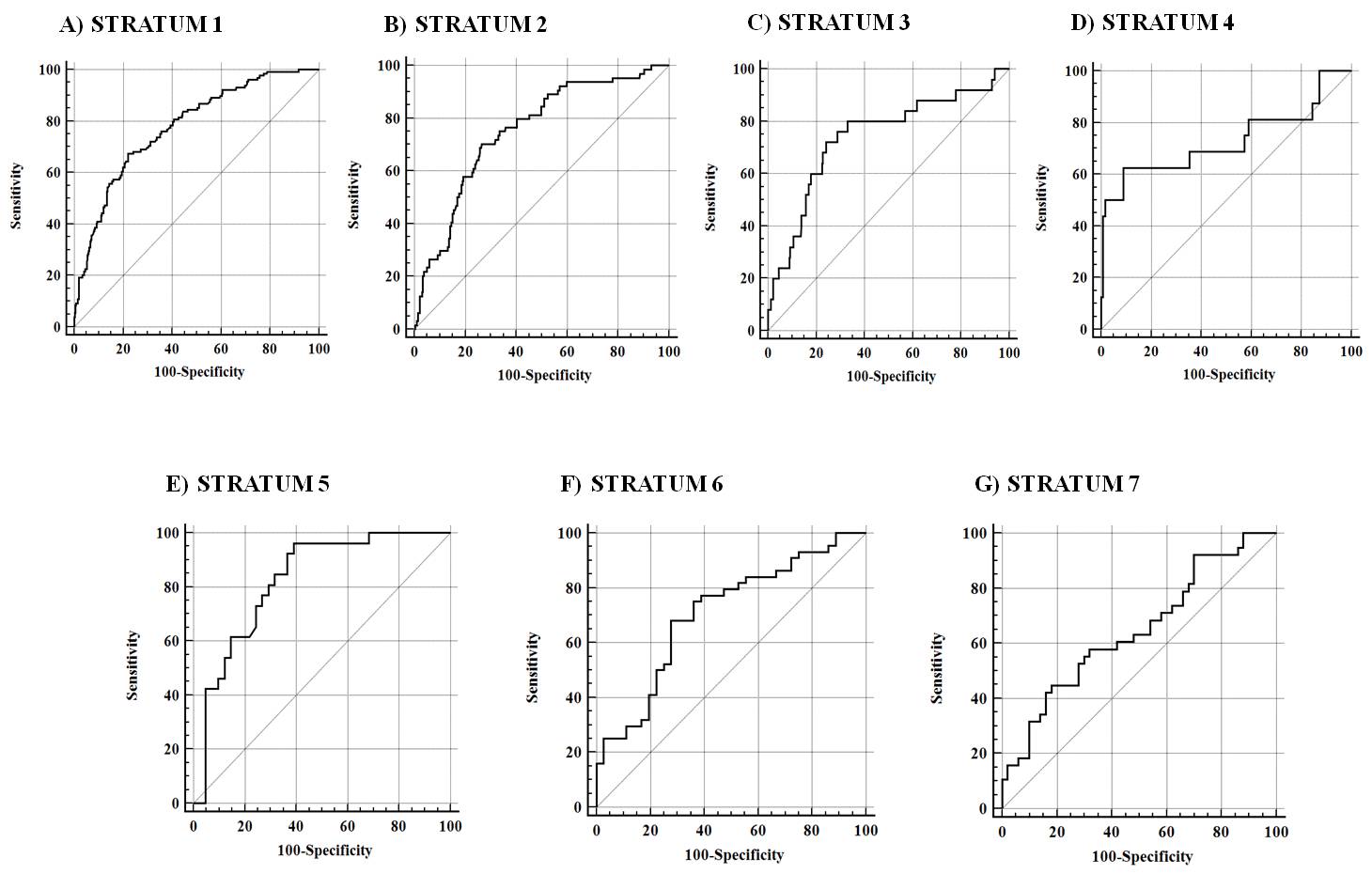
**

| **Stratum (baseline eGFR)** | **Sensitivity (%)** | **Specificity (%)** | **Positive predictive value (%)** | **Negative predictive value (%)** |
| --- | --- | --- | --- | --- |
| **1 (≥80 ml/min/1.73 m^2^)** | 67.4 | 78.0 | 53.0 | 86.7 |
| **2 (70-79 ml/min/1.73 m^2^)** | 70.3 | 73.5 | 34.9 | 92.5 |
| **3 (60-69 ml/min/1.73 m^2^)** | 72.0 | 75.8 | 24.0 | 96.2 |
| **4 (50-59 ml/min/1.73 m^2^)** | 62.5 | 90.9 | 50.0 | 94.3 |
| **5 (40-49 ml/min/1.73 m^2^)** | 96.2 | 61.0 | 61.0 | 96.2 |
| **6 (30-39 ml/min/1.73 m^2^)** | 68.2 | 72.2 | 75.0 | 65.0 |
| **7 (<30 ml/min/1.73 m^2^)** | 44.7 | 82.0 | 65.4 | 66.1 |

**SUPPLEMENTARY FIGURE 3**

**
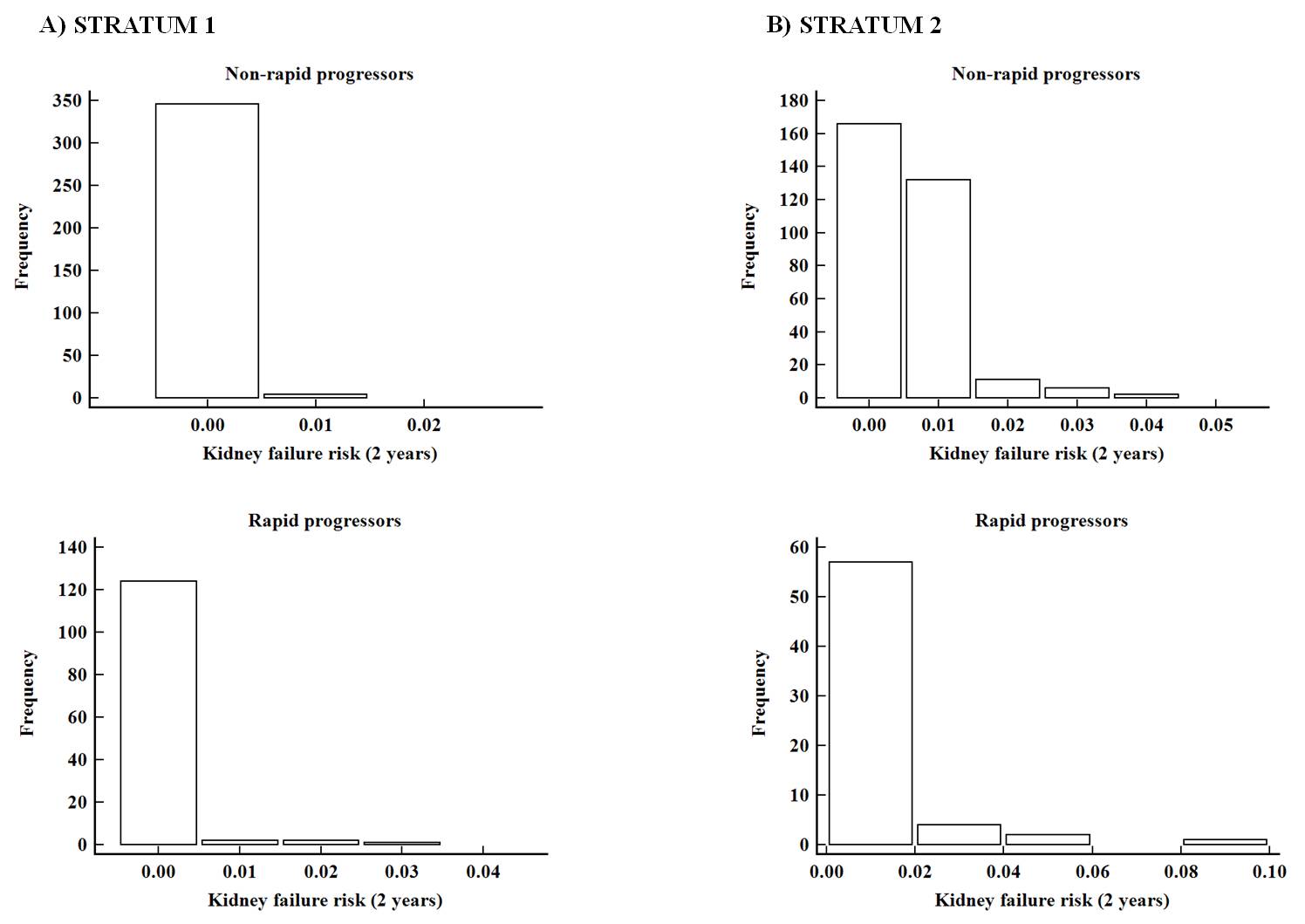
**

**
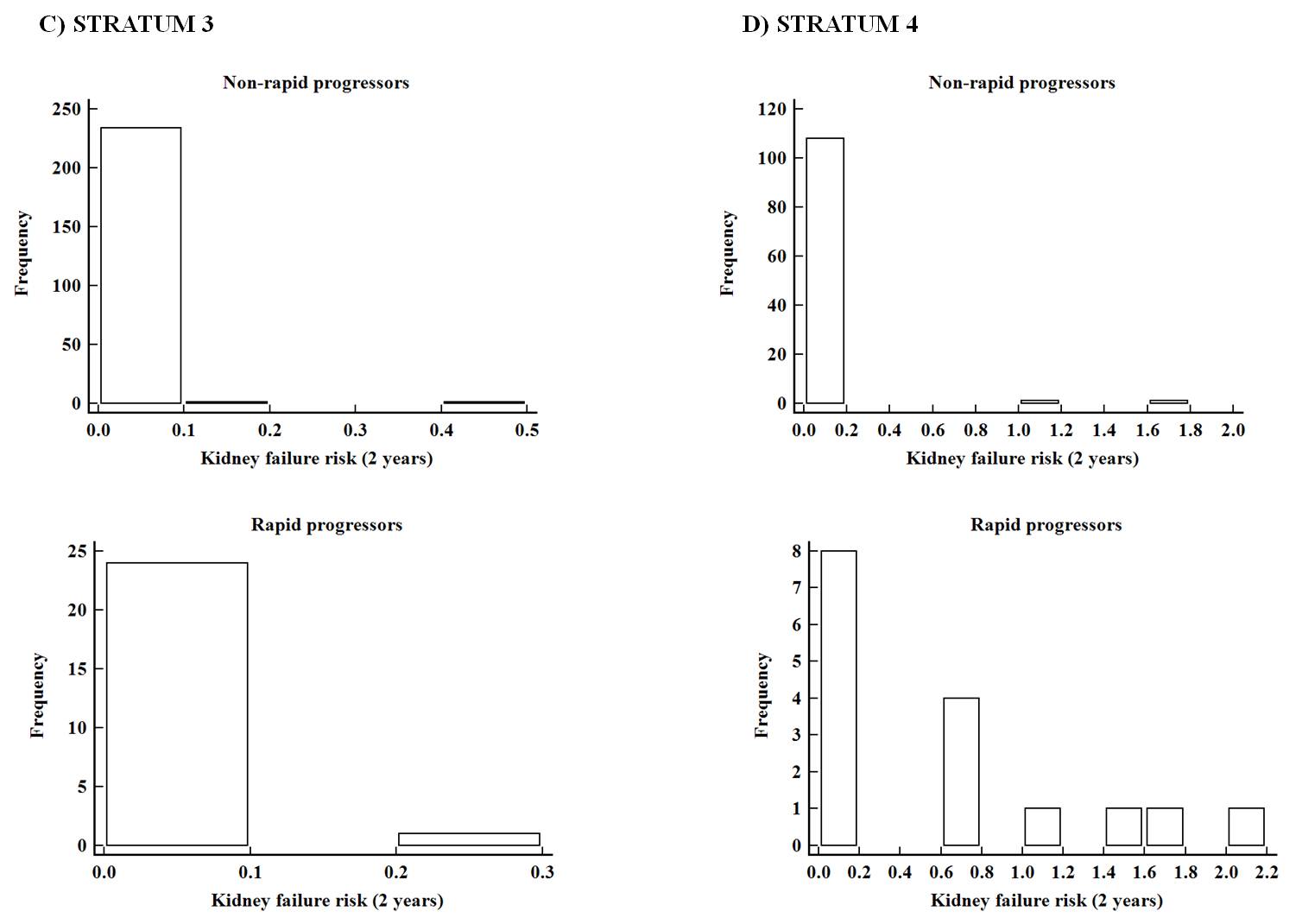
**

**
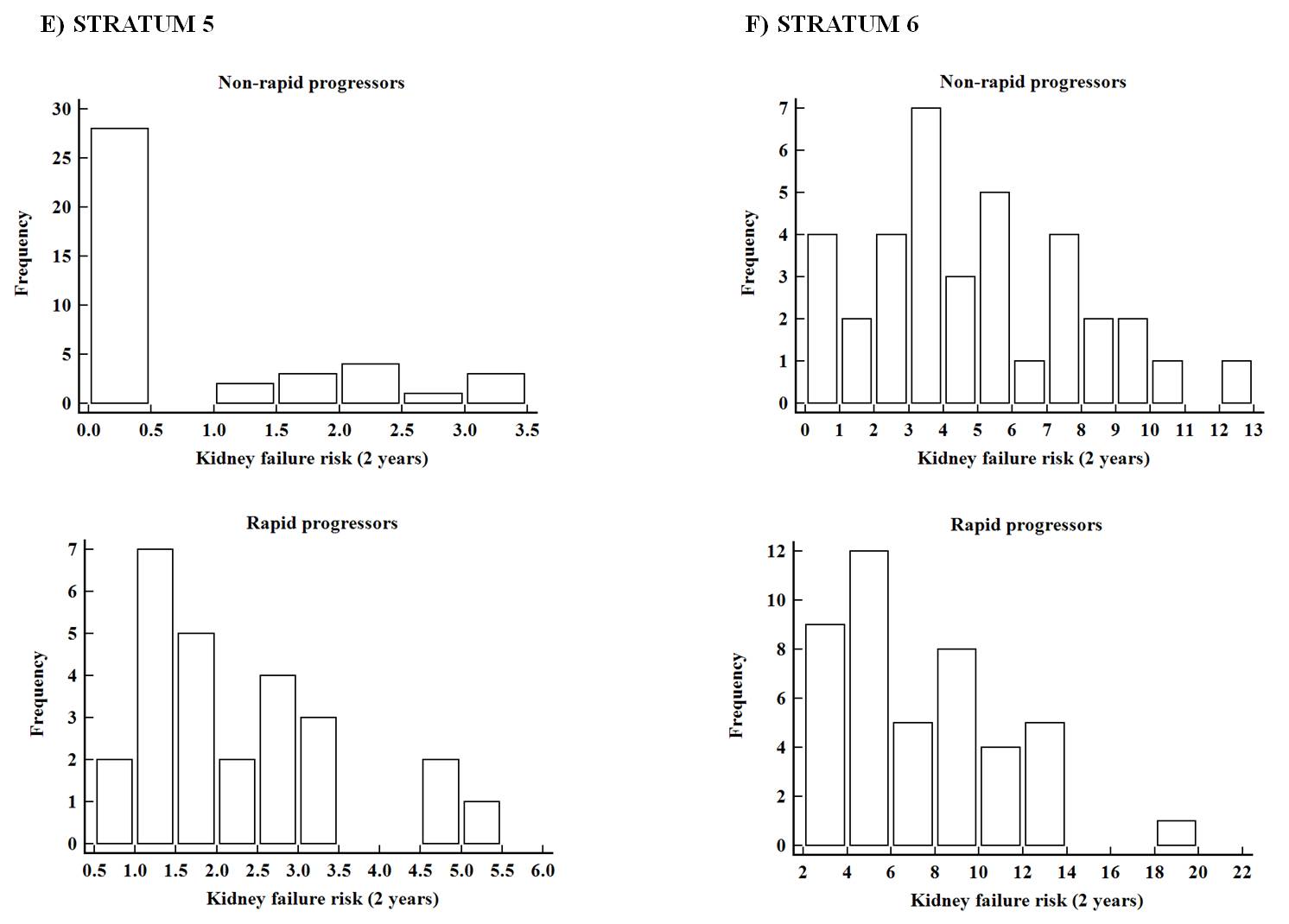
**

**
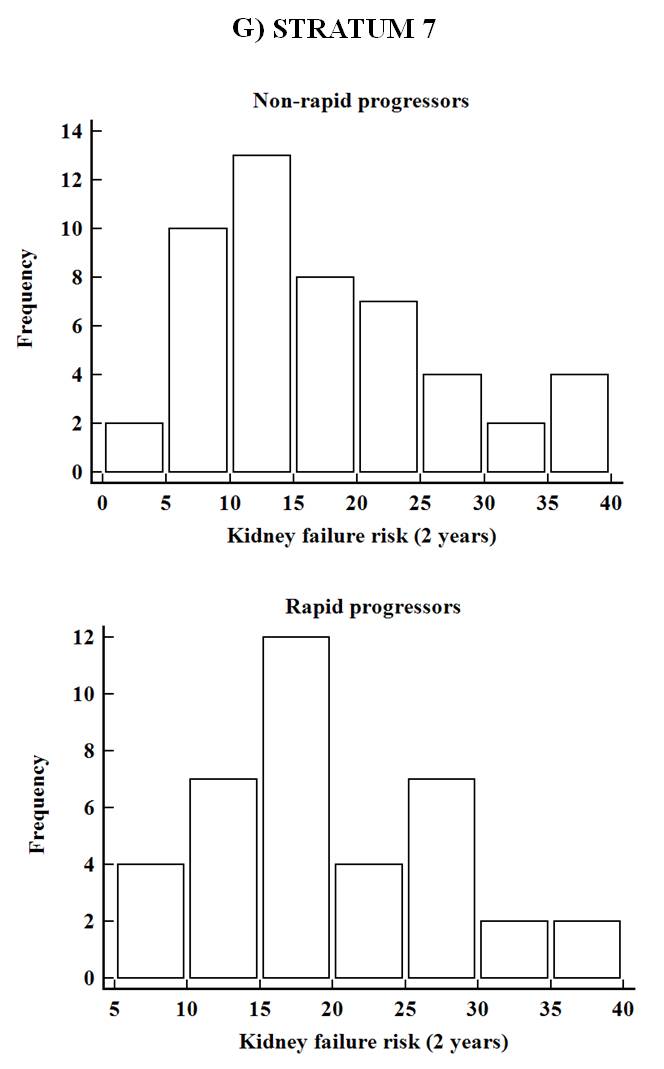
**

|  | **Rapid progressors** | | | **Non-rapid progressors** | | |
| --- | --- | --- | --- | --- | --- | --- |
| **Stratum (baseline eGFR)** | **Q25** | **Q50** | **Q75** | **Q25** | **Q50** | **Q75** |
| **1 (≥80 ml/min/1.73 m^2^)** | 0.000 | 0.000 | 0.000 | 0.000 | 0.000 | 0.000 |
| **2 (70-79 ml/min/1.73 m^2^)** | 0.000 | 0.000 | 0.010 | 0.000 | 0.000 | 0.010 |
| **3 (60-69 ml/min/1.73 m^2^)** | 0.010 | 0.020 | 0.035 | 0.010 | 0.020 | 0.030 |
| **4 (50-59 ml/min/1.73 m^2^)** | 0.050 | 0.410 | 1.020 | 0.040 | 0.050 | 0.070 |
| **5 (40-49 ml/min/1.73 m^2^)** | 1.245 | 1.960 | 2.902 | 0.110 | 0.200 | 1.785 |
| **6 (30-39 ml/min/1.73 m^2^)** | 4.200 | 6.705 | 9.930 | 2.745 | 4.590 | 7.525 |
| **7 (<30 ml/min/1.73 m^2^)** | 14.020 | 17.615 | 25.592 | 10.312 | 14.890 | 23.610 |

**SUPPLEMENTARY FIGURE 4**

**
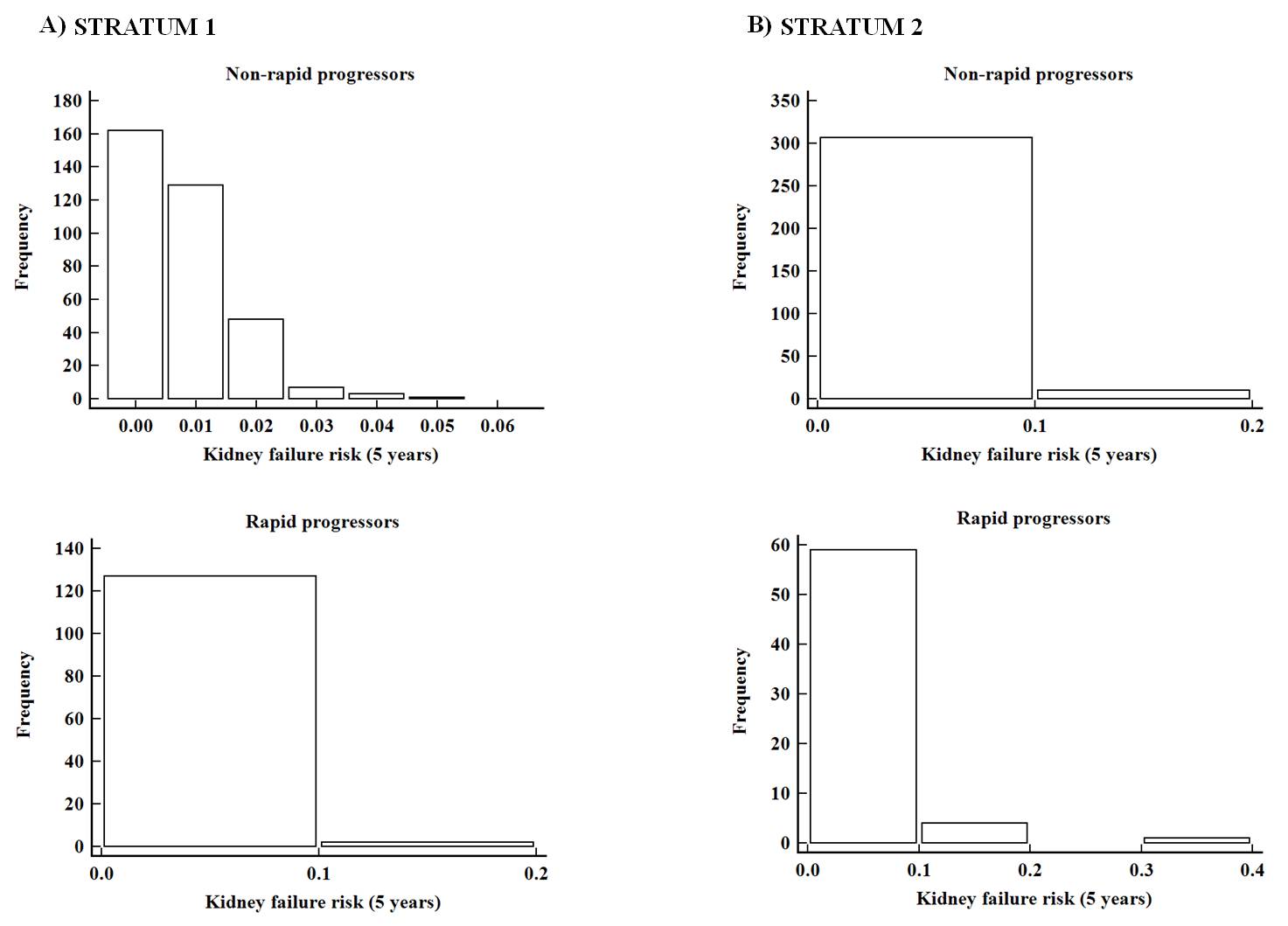
**

**
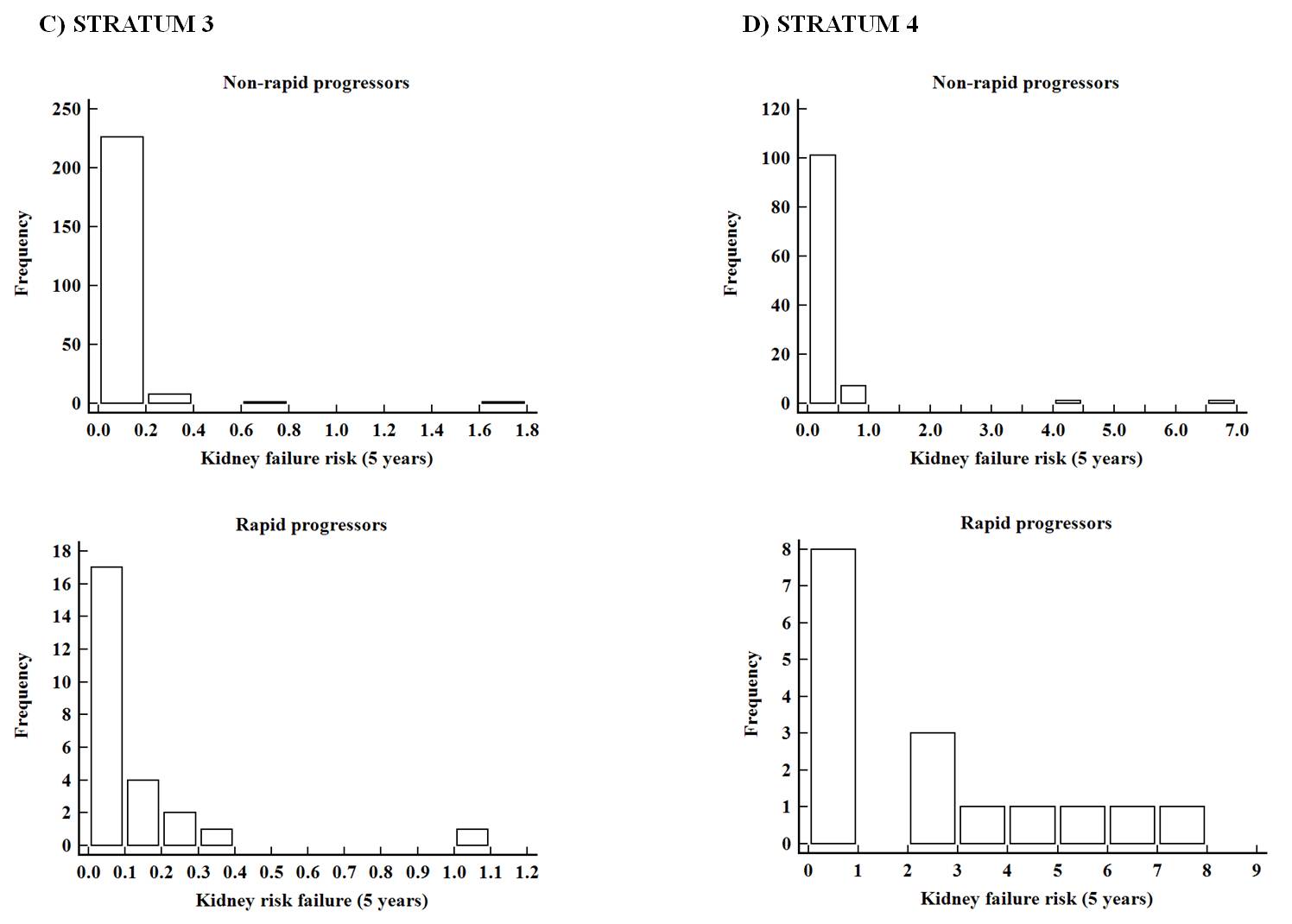
**

**
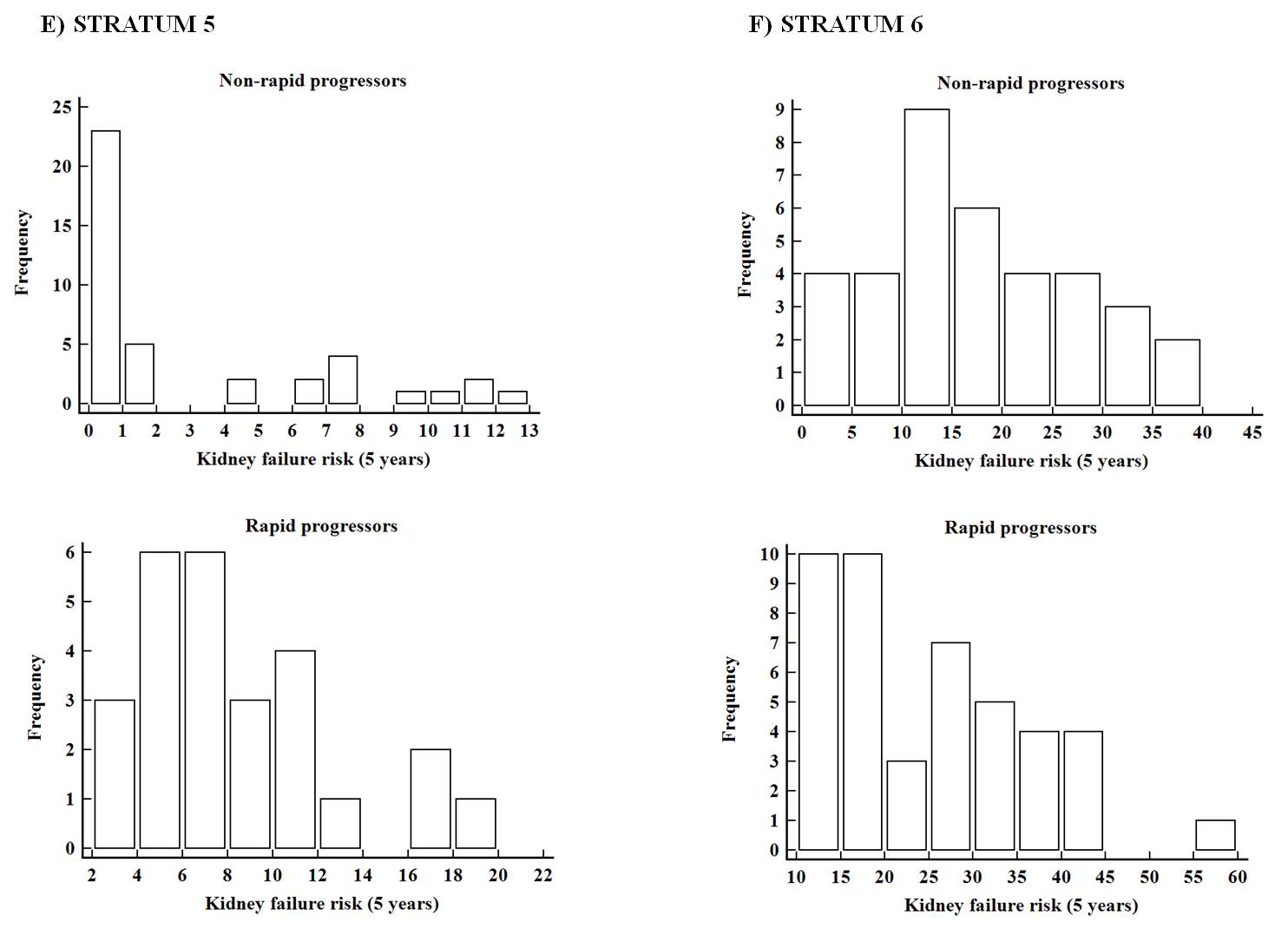
**

**
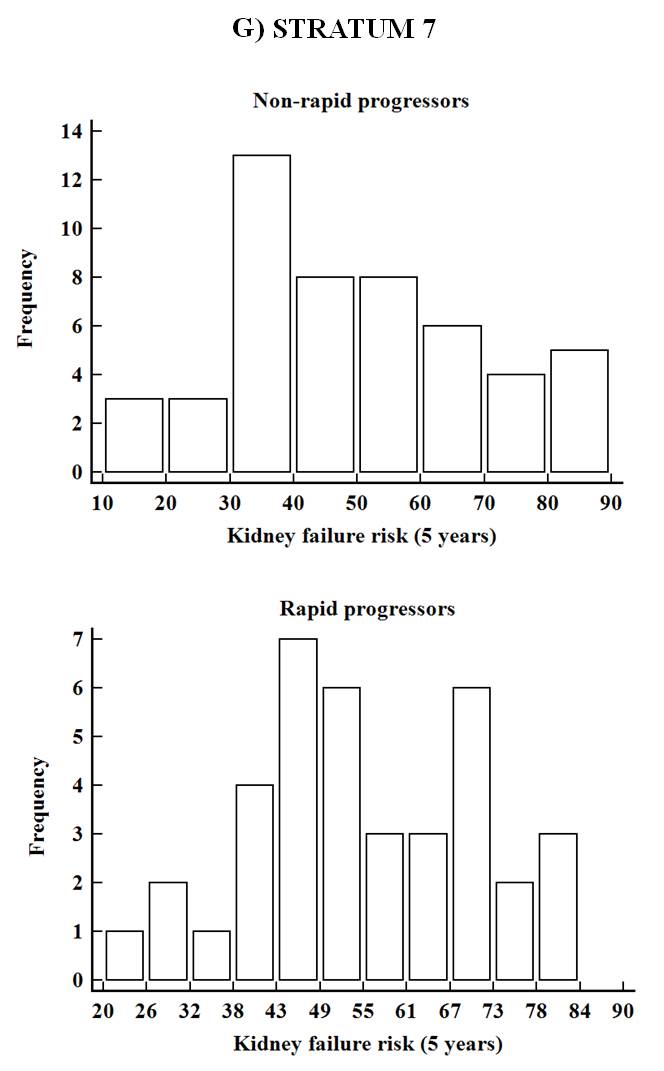
**

|  | **Rapid progressors** | | | **Non-rapid progressors** | | |
| --- | --- | --- | --- | --- | --- | --- |
| **Stratum (baseline eGFR)** | **Q25** | **Q50** | **Q75** | **Q25** | **Q50** | **Q75** |
| **1 (≥80 ml/min/1.73 m^2^)** | 0.000 | 0.000 | 0.010 | 0.000 | 0.010 | 0.010 |
| **2 (70-79 ml/min/1.73 m^2^)** | 0.010 | 0.020 | 0.040 | 0.020 | 0.030 | 0.050 |
| **3 (60-69 ml/min/1.73 m^2^)** | 0.050 | 0.070 | 0.140 | 0.060 | 0.080 | 0.110 |
| **4 (50-59 ml/min/1.73 m^2^)** | 0.192 | 1.590 | 3.895 | 0.150 | 0.200 | 0.270 |
| **5 (40-49 ml/min/1.73 m^2^)** | 4.752 | 7.375 | 10.800 | 0.440 | 0.790 | 6.765 |
| **6 (30-39 ml/min/1.73 m^2^)** | 15.332 | 23.570 | 33.305 | 10.232 | 16.645 | 26.132 |
| **7 (<30 ml/min/1.73 m^2^)** | 44.290 | 52.775 | 68.177 | 34.395 | 46.440 | 64.755 |

**SUPPLEMENTARY FIGURE 5**

**
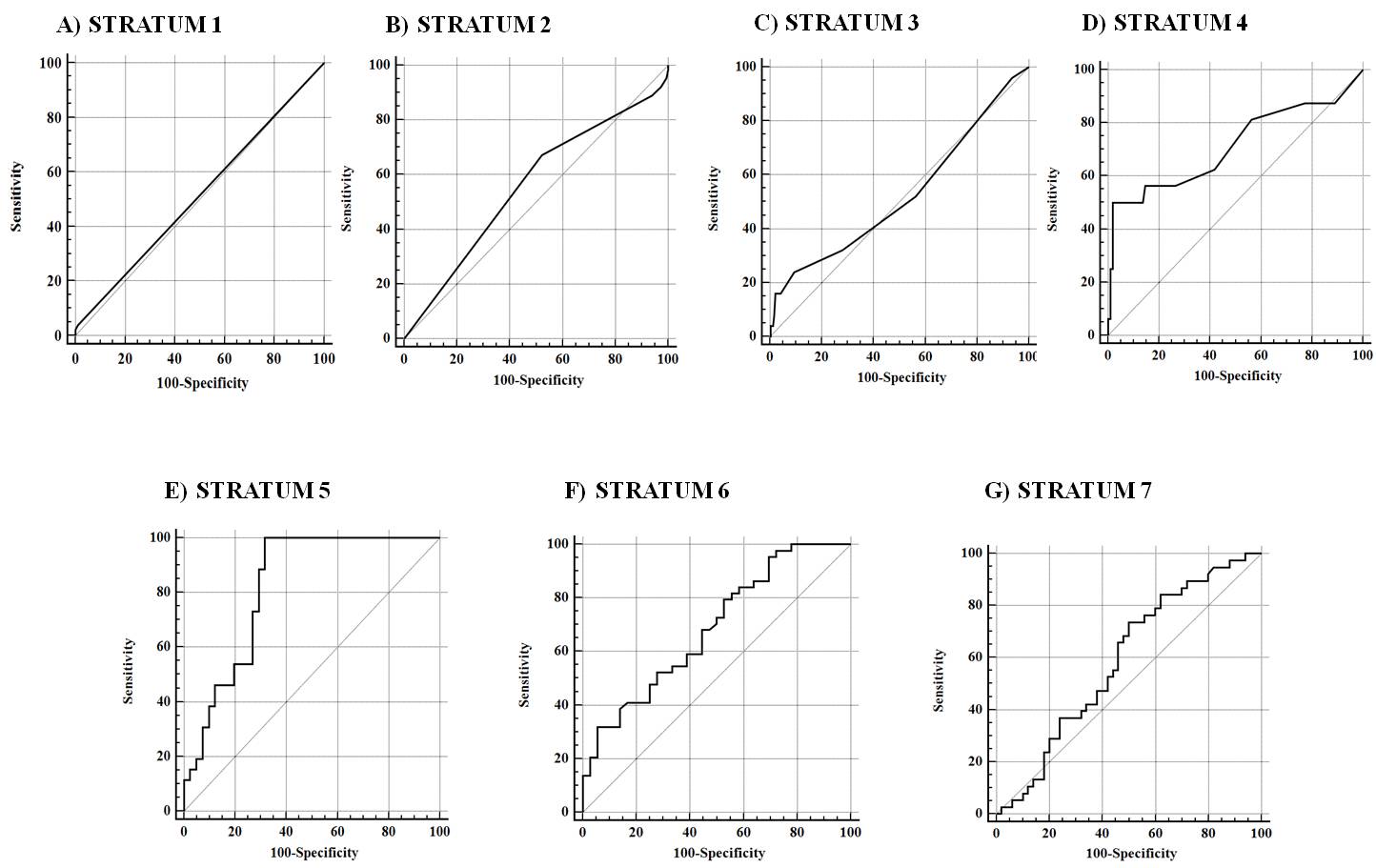
**

| **Stratum (baseline eGFR)** | **Sensitivity (%)** | **Specificity (%)** | **Positive predictive value (%)** | **Negative predictive value (%)** |
| --- | --- | --- | --- | --- |
| **1 (≥80 ml/min/1.73 m^2^)** | 3.9 | 98.9 | 55.6 | 73.6 |
| **2 (70-79 ml/min/1.73 m^2^)** | 67.2 | 47.6 | 20.6 | 87.2 |
| **3 (60-69 ml/min/1.73 m^2^)** | 24.0 | 90.7 | 21.4 | 91.8 |
| **4 (50-59 ml/min/1.73 m^2^)** | 50.0 | 98.2 | 80.0 | 93.1 |
| **5 (40-49 ml/min/1.73 m^2^)** | 100.0 | 68.3 | 66.7 | 100.0 |
| **6 (30-39 ml/min/1.73 m^2^)** | 79.5 | 47.2 | 64.8 | 65.4 |
| **7 (<30 ml/min/1.73 m^2^)** | 73.7 | 50.0 | 52.8 | 71.4 |

**SUPPLEMENTARY FIGURE 6**

**
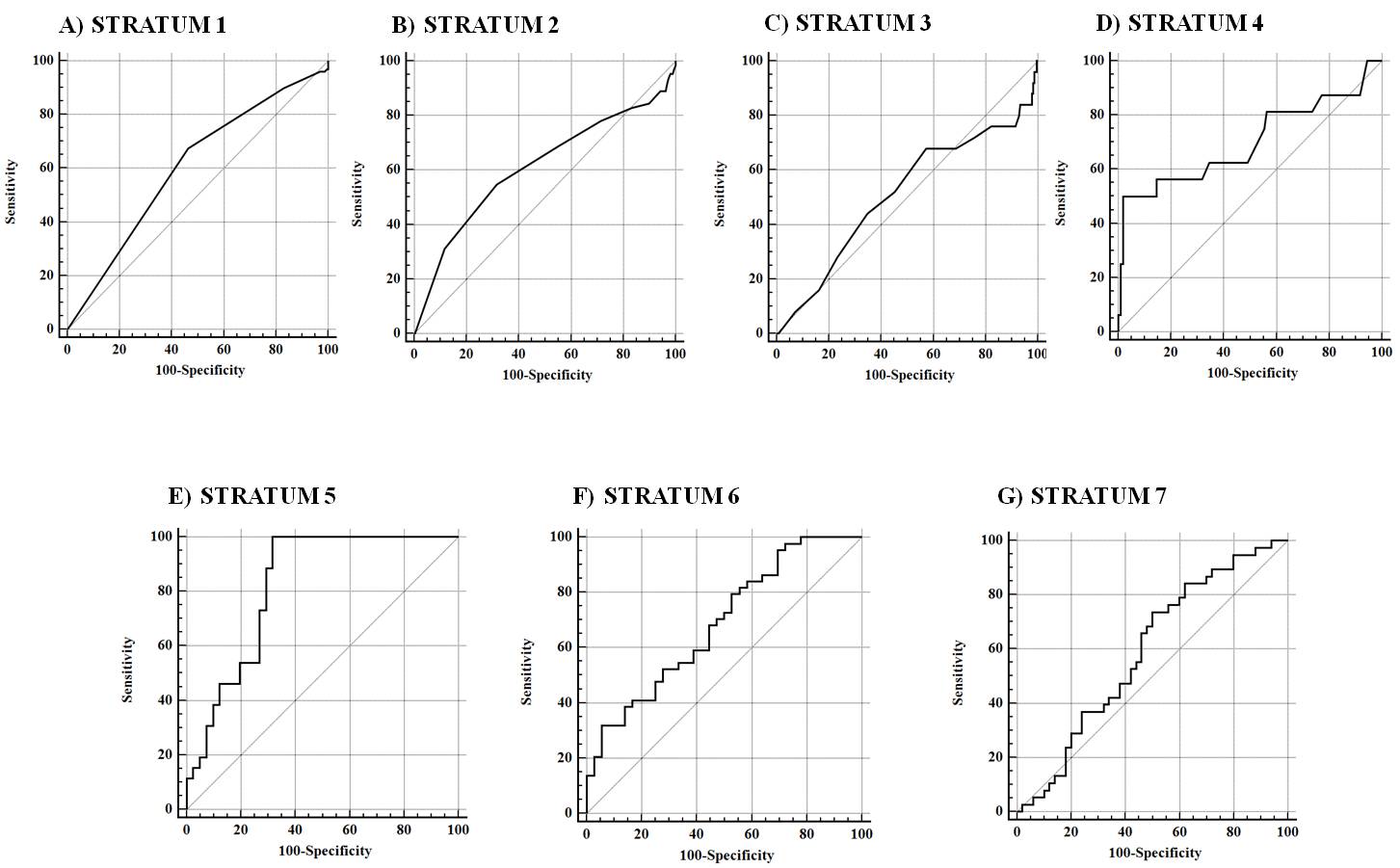
**

| **Stratum (baseline eGFR)** | **Sensitivity (%)** | **Specificity (%)** | **Positive predictive value (%)** | **Negative predictive value (%)** |
| --- | --- | --- | --- | --- |
| **1 (≥80 ml/min/1.73 m^2^)** | 67.4 | 53.7 | 34.9 | 81.7 |
| **2 (70-79 ml/min/1.73 m^2^)** | 54.7 | 68.5 | 25.9 | 88.2 |
| **3 (60-69 ml/min/1.73 m^2^)** | 76.0 | 8.5 | 8.1 | 76.9 |
| **4 (50-59 ml/min/1.73 m^2^)** | 50.0 | 98.2 | 80.0 | 93.1 |
| **5 (40-49 ml/min/1.73 m^2^)** | 100.0 | 68.3 | 66.7 | 100.0 |
| **6 (30-39 ml/min/1.73 m^2^)** | 79.5 | 47.2 | 64.8 | 65.4 |
| **7 (<30 ml/min/1.73 m^2^)** | 73.7 | 50.0 | 52.8 | 71.4 |
